# Identification of Novel Alternative Transcripts of the Human ALKBH Gene Family and Investigation of Their Unique Expression Signatures in Cancer Cells

**DOI:** 10.3390/cimb48030251

**Published:** 2026-02-26

**Authors:** Konstantina Athanasopoulou, Vasiliki-Ioanna Michalopoulou, Panagiotis Tsiakanikas, Andreas Scorilas, Panagiotis G. Adamopoulos

**Affiliations:** Department of Biochemistry and Molecular Biology, Faculty of Biology, National and Kapodistrian University of Athens, 15701 Athens, Greece; konnath@biol.uoa.gr (K.A.); vimich@biol.uoa.gr (V.-I.M.); ptsiak@biol.uoa.gr (P.T.); ascorilas@biol.uoa.gr (A.S.)

**Keywords:** alternative splicing, demethylases, ALKBH, third-generation sequencing, direct RNA nanopore sequencing, qPCR, epigenetic modifications

## Abstract

The human *ALKBH* gene family comprises nine Fe^2+^/α-ketoglutarate-dependent dioxygenases that catalyze the oxidative demethylation of DNA, RNA, and proteins, thereby influencing key cellular processes. Consequently, dysregulation of these enzymes has been implicated in various human diseases, particularly cancer. Although the transcriptomic profiles of certain members (e.g., *ALKBH8*, *FTO*) have been characterized, a comprehensive analysis of the entire *ALKBH* family remains unclear. In the present study, we investigated the alternative splice variants of the *ALKBH* genes through direct RNA sequencing across cancerous and non-cancerous cell lines. Novel splicing events were validated by NGS, while RT-qPCR was employed to assess transcript abundance and expression patterns. Additionally, in silico analysis was performed to predict the coding potential of the detected transcripts. Results: Bioinformatics analysis revealed previously uncharacterized alternative transcripts for the human *ALKBH* gene family members. Expression profiling demonstrated distinct expression patterns between cancerous and non-malignant cells, suggesting a potential role of these demethylases in tumor biology. The investigation of their coding capacity revealed that most of the newly detected transcripts were predicted to encode protein isoforms, highlighting the structural and predicted coding potential of the ALKBH family. Conclusions: Our findings provide the first comprehensive overview of the transcriptional diversity within the human *ALKBH* gene family. These results enhance our understanding of the demethylation mechanisms and their dysregulation in cancer.

## 1. Introduction

The field of epigenetic modifications has emerged as a transformative area in molecular biology, advancing our understanding of reversible modifications that occur in DNA, RNA and proteins, as well as their regulatory roles. Central to this paradigm is the methylation of nucleic acids, histones and other proteins, which is a reversible alteration proven to influence gene expression and other biological procedures [1]. This modification is dynamically regulated by a variety of enzymes, including the ALKBH family, a group of nine dioxygenases that remove methyl groups by oxidative demethylation, influencing key cellular processes, such as repair of alkylation damage in nucleic acid and nucleoprotein complexes [2]. Specifically, in RNAs, ALKBH5 has been shown to demethylate m6A marks, while ALKBH1 and ALKBH3 are involved in the demethylation of m1A and/or m3C, thereby influencing DNA/RNA metabolism [3,4,5]. Additionally, ALKBH1 and ALKBH4 have been found to remove methyl adducts from H2A and actin, respectively [6,7]. Notably, the activity of some ALKBH enzymes, including ALKBH6, has not yet been elucidated.

Accumulating evidence suggests that the dysregulation of ALKBH enzymes is closely associated with human diseases, particularly cancer. Several studies have reported altered expression patterns of *ALKBH* genes in a variety of tumor types, suggesting their potential role in tumorigenesis [8]. Notably, ALKBH5 has been found to be upregulated in Triple-Negative Breast Cancer (TNBC), highlighting its potential role as a biomarker or therapeutic target for cancer treatment [9,10]. Furthermore, single-cell analyses have uncovered links between *ALKBH1* expression and tumor-associated macrophages in gastric cancer, suggesting that ALKBH1 may participate in the tumor microenvironment and immune response modulation [11]. Accordingly, studies on head and neck cancer suggest that overexpression of ALKBH enzymes may also serve as potential targets for anticancer therapies [12]. Although the precise mechanisms remain to be fully clarified, these findings highlight the need for further exploration of the role of *ALKBH* genes in cancer biology.

Beyond their well-established epitranscriptomic roles, *ALKBH* genes are also subjected to complex post-transcriptional regulations, including alternative splicing. This conserved cellular process enables the generation of multiple mRNA isoforms from a single gene, thereby enhancing transcriptomic diversity and allowing cells to finely modulate protein function [13,14]. Notably, the production of alternative transcripts is essential for the regulation of gene expression and cellular adaptation. However, our understanding of alternative splicing events within the *ALKBH* gene family remains limited. Although alternative mRNA variants have been identified in certain *ALKBH* genes, such as *ALKBH8* and the closely related FTO, the full spectrum of splice variants across the entire *ALKBH* gene family has yet to be comprehensively characterized [15].

In this study, we employed direct RNA nanopore sequencing (dRNA-seq) to investigate alternative splicing events within the *ALKBH* gene family across various human cell lines. By analyzing full-length RNA sequences, our approach provides a comprehensive view of transcript diversity, overcoming the limitations of short-read sequencing methods. Our findings reveal that alternative splicing of *ALKBH* genes is significantly associated with, and distinguishes between, multiple cancer types. Notably, distinct expression patterns of specific *ALKBH* isoforms suggest their potential as biomarkers for disease prognosis. In addition, we experimentally tested the open reading frames (ORFs) of novel isoforms and performed in silico analyses to predict potential protein-coding capacities or characterize the transcripts as long non-coding RNAs (lncRNAs). Together, these results uncover novel links between *ALKBH* splicing variations and cancer pathogenesis, offering new insights into the regulatory mechanisms governing these critical demethylases.

## 2. Materials and Methods

### 2.1. Biological Material

In this study, we investigated alternative splicing events in members of the ALKBH gene family across a panel of human cell lines (Supplementary Table S1). For this purpose, we cultured cancer-derived cell lines, including, BT-20, HCT-116, HeLa, HepG2, OVCAR3, PC-3, RT-112, THP-1 and U-2 OS, along with the non-tumorigenic cell lines HEK-293 and MCF 10A. All cell lines were cultured according to the standard protocols and media formulations provided by the American Type Culture Collection (ATCC).

### 2.2. Total RNA Extraction and Poly(A) RNA Enrichment

Total RNA was extracted from all human cell lines using TRIzol Reagent (Ambion™, Thermo Fisher Scientific Inc., Waltham, MA, USA). To enrich the polyadenylated mRNAs, the Magnosphere™ UltraPure mRNA Purification Kit (Takara Bio Inc.) was used according to the manufacturer’s protocol. The concentration and integrity of both total RNA and purified mRNA were assessed using Qubit fluorometric quantification (Thermo Fisher Scientific) and capillary electrophoresis on the Qsep system (BiOptic Inc.), ensuring high-quality input for downstream applications.

### 2.3. Library Preparation and Direct RNA Sequencing

dRNA-seq was performed on HeLa, BT-20, and MCF 10A cell lines. For library preparation, 500 ng of mRNA was used as the starting material. Sequencing libraries were constructed using the Direct RNA sequencing kit (SQK-RNA002, Oxford Nanopore Technologies Inc., ONT), following the manufacturer’s instructions. Briefly, the RT Adapter (RTA) was annealed and ligated to the 3′ poly(A) tails of mRNAs by incubating each sample at room temperature for 10 min using T4 DNA ligase (New England Biolabs Inc., Ipswich, MA, USA). Reverse transcription was then performed in a 40 μL reaction volume using a hot-lid Veriti™ 96-Well Fast Thermal Cycler (Applied Biosystems™). Samples were purified with 1.8X Agencourt RNAClean XP beads (Beckman Coulter Inc.). Subsequently, the RNA Adapter (RMX) was ligated to the cDNAs by incubating for 10 min at room temperature with T4 DNA ligase, followed by a second purification step. Each library was loaded onto a FLO-MIN106D flow cell with R9.4.1 chemistry, and sequencing was performed on MinION Mk1C sequencer (Oxford Nanopore Technologies Ltd., ONT).

### 2.4. Bioinformatics Processing of Sequencing Data

To extend and strengthen our study, we also integrated publicly available RNA sequencing datasets from the Sequence Read Archive (SRA). These datasets originated from previously published nanopore direct RNA sequencing experiments and included multiple human cell lines, such as A549, Huh7, HepG2, K562, U-2 OS, and HEK-293.

To ensure consistency and minimize bias during data analysis, all sequencing datasets—both experimentally generated in our laboratory and public—were processed using identical bioinformatics pipelines, employing the same algorithm versions and parameters. Raw sequencing data were basecalled using Guppy v.6.2.1, with the RNA model rna_r9.4.1_70bps_hac and a default read Q-score filtering threshold of 7. Only reads that passed the quality filtering step were retained for further analysis. Basecalled reads were aligned to human reference genome (NCBI RefSeq assembly: GCF_000001405.40) using minimap2 (version 2.22) with the “-ax splice -uf -k14” enabled. The aligned reads were visualized using Integrative Genomics Viewer (IGV) [16,17]. Expression analysis, transcript quantification and the detection of novel mRNAs within the *ALKBH* gene family were conducted with IsoQuant v.3.3.1 and the default run parameters for nanopore sequencing [18].

### 2.5. Next-Generation Sequencing

Standard RNA-seq was conducted to validate the splicing events detected by nanopore sequencing, using a different chemistry of NGS. For this purpose, 10 ng of poly(A)+ RNA extracted from HEK-293 cells was used to construct sequencing libraries, following the protocol provided with the MGIEasy RNA Directional Library Prep Set kit (MGI Tech Co., Ltd.). Sequencing was performed on the DNBSEQ-G50 platform (MGI Tech Co., Ltd.) using an FCL flow cell and paired-end 100 bp (PE100) read configuration.

To extend our analysis and provide additional context, two publicly available short-read RNA-seq datasets were obtained from SRA: one from a TRAP-Seq study on human pluripotent stem cells (Study ID: PRJNA687244), and the other from a Poly-Ribo-Seq study performed on HEK-293T cells (Study ID: PRJNA853906). All short-read sequencing data were aligned to the human reference genome (GRCh38.p14) using the HISAT2 with default parameters [19].

### 2.6. RT-qPCR Profiling of ALKBH mRNA Abundance

The relative abundance of *ALKBH* mRNAs was assessed by quantitative real-time PCR (RT-qPCR) using variant-specific primers designed to target novel splice junctions of the *ALKBH* genes (Supplementary Table S2). The qPCR assays were performed using cDNA samples derived from various human cell lines, as outlined in Supplementary Table S1. For cDNA synthesis, 5 μg of total RNA served as the template, and reverse transcription (RT) was performed using an oligo-dT primer. A 14.5 μL reaction mix was prepared, containing the total RNA, 1 μL of oligo-dT (100 pmol), and 1 μL of dNTP mix (10 mM each). This mixture was incubated in a Veriti 96-Well Fast Thermal Cycler (Applied Biosystems™) with a heated lid at 65 °C for 5 min, followed by cooling on ice for 2 min. The total volume was then brought to 20 μL by adding 4 μL of 5X RT Buffer, 20 U RiboLock RNase Inhibitor and 200 U of Maxima Reverse Transcriptase (Invitrogen™, Thermo Fisher Scientific Inc.). The RT reaction was performed at 50 °C for 30 min and terminated by heating the mixture to 85 °C for 5 min. Then, cDNA was diluted 1:50 in nuclease-free H_2_O and used as template for qPCR.

qPCR assays were conducted using the QuantStudio™ 5 Real-Time PCR System (Applied Biosystems™). Each reaction was performed in a 10 μL volume, containing 5 μL of 2X Kapa SYBR™ Fast qPCR Master Mix (KapaBiosystems, Inc.), 2 μM of each primer, and 1 μL of cDNA template. The thermal cycling conditions included an initial denaturation step at 95 °C for 3 min, followed by 40 cycles of denaturation at 95 °C for 3 s and annealing at 60 °C for 30 s. The human glyceraldehyde-3-phosphate dehydrogenase (*GAPDH*) gene was used as an endogenous reference control for normalization of data.

### 2.7. Data Analysis

Statistical analysis of the qPCR data was performed using GraphPad Prism 9 (GraphPad Software ver. 9, San Diego, CA, USA). The results are expressed as mean ± standard deviation (SD). To compare the expression levels of ALKBH variants, multiple unpaired *t*-tests were employed, assuming a Gaussian distribution. All statistical tests were performed on three technical replicates, and statistical significance was set at *p* < 0.05. Error bars represent the standard deviation of the means.

### 2.8. In Silico Structural Analysis of Alternative ALKBH Isoforms

Standard To investigate the functional potential of alternative *ALKBH* transcripts, both protein-coding and non-coding capacities were systematically analyzed. The ExPASy webserver was first utilized to assess the protein-coding potential of each transcript by identifying annotated and putative start and stop codons and translating the mRNA sequences into their corresponding amino acid sequences [20]. These predicted protein sequences were then examined for the presence of conserved ALKBH motifs, providing further evidence of their potential to encode functionally relevant isoforms.

To explore the biological significance of these predicted proteins, functional annotation was performed using the DeepGOWeb Protein Function Prediction server [21]. This analysis yielded Gene Ontology (GO) enrichment results, along with confidence scores, across the two GO terms: Molecular Function and Biological Process. Additionally, protein isoforms were aligned using the UniProt Clustal Omega alignment tool via the “Align” function. We selected the “Clustal” highlight mode to visualize amino acid conservation based on residue chemical properties (Supplementary Data). In this mode, residues are colored according to Clustal/ClustalX conventions: charged residues (K, R, D, E, H) in red, aromatic residues (F, Y, W) in blue, hydrophobic aliphatic residues (M, L, I, V, C) in green, small/polar/helix-disrupting residues (G, P, T, S, A) in orange, and polar uncharged residues (e.g., N, Q) in white. The annotated alignment was exported and used to identify conserved motifs, active-site residues, and structural differences between isoforms. Subsequently, AlphaFold3 was employed to predict the three-dimensional structures of the putative protein isoforms [22]. Structural alignment and comparison with annotated ALKBH proteins were performed using PyMOL.5.

## 3. Results

### 3.1. Nanopore Sequencing Unveils the Existence of ALKBH1 v.2

**Figure 1:**
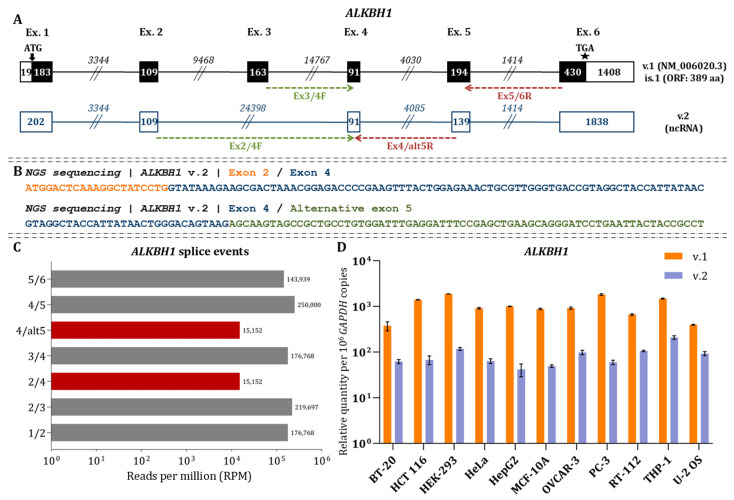
Characterization of the *ALKBH1* transcript variants. (**A**) Schematic demonstration of the *ALKBH1* mRNAs. Exons are shown as boxes and introns as lines. Black boxes indicate ORFs, while white boxes denote UTRs. Numbers within boxes and above lines correspond to nucleotide length of exons and introns, respectively. Arrows mark the ATG, and asterisks (★) indicate the termination codon. The transcript number and GenBank^®^ accession number are shown adjacent to each transcript. Green and red arrows denote the relative positions of the forward and reverse primers used for mRNA amplification. (**B**) NGS sequencing reads confirming the exon-skipping event that characterizes the newly identified *ALKBH1* v.2. (**C**) Quantification of annotated and novel *ALKBH1* splicing events based on RPM values. (**D**) Relative expression levels of *ALKBH1* variants. Bar plots show the abundance of each variant using variant-specific primers. *GAPDH* was used as the housekeeping gene for normalization. Data are expressed as *ALKBH1* mRNA copies per 10^6^ *GAPDH* copies. All qPCR experiments were performed in triplicate using independent biological replicates.

dRNA-seq confirmed the presence of the annotated *ALKBH1* transcript (v.1: NM_006020.3), which comprises six exons and encodes a 389-amino-acid protein. In addition, we identified a previously unannotated alternative transcript, which was named *ALKBH1* v.2 (GenBank^®^ accession number: PX620489). *ALKBH1* v.2 is characterized by an alternative splice junction between exons 2 and 4 and a truncated 5′ region of exon 5 (Figure 1A).

Although ALKBH1 v.2 retains the canonical initiation codon, it contains a premature termination codon (PTC), suggesting that it is either subject to nonsense-mediated decay (NMD) or functions as a lncRNA. The presence of the alternative splice junction between exons 2 and 4 was independently validated by short-read RNA sequencing, which yielded NGS reads spanning this novel junction (Figure 1B). Quantitative analysis of the NGS data revealed that the abundance of this novel splicing event was markedly lower than that of the splice junctions defining the main ALKBH1 transcript (Figure 1C). Furthermore, RT-qPCR assay was conducted using specific primers designed to target the splice junction 4/6. Results showed that ALKBH1 v.2 was significantly underrepresented compared to the main ALKBH1 across all examined cell lines (*p* < 0.05; Figure 1D), consistent with the RNA-seq data.

### 3.2. Identification of a Novel Transcript ALKBH2 v.6

**Figure 2:**
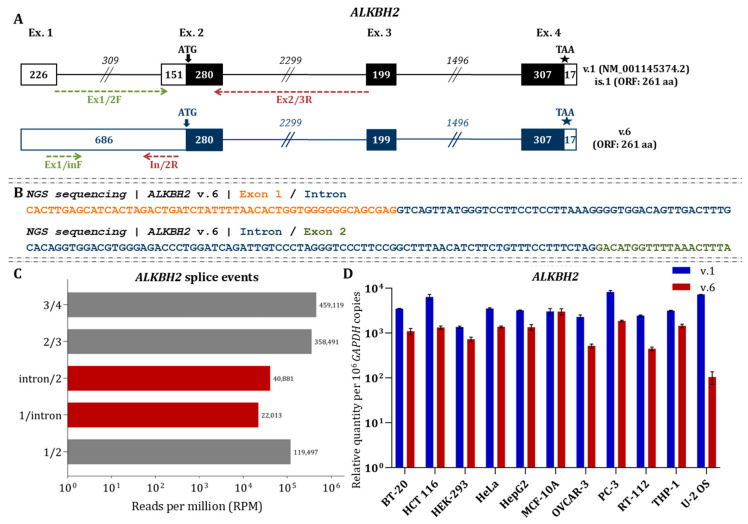
Bioinformatics and expression analysis of *ALKBH2* mRNAs. (**A**) Schematic representation of the two *ALKBH2* splice variants. Exons are depicted as boxes and introns as connecting lines. Black boxes indicate coding regions, while white boxes denote UTRs. Numbers within boxes and above lines correspond to exon and intron lengths, respectively. Arrows mark the initiation codon, and asterisks (★) indicate the termination codon. The GenBank^®^ accession numbers are shown adjacent to each transcript. Green and red arrows denote the relative positions of the forward and reverse primers used for mRNA amplification. (**B**) Representative NGS sequencing reads validating the intron retention that characterizes the newly identified *ALKBH2* mRNA. (**C**) RPM values illustrating the abundance of annotated and novel *ALKBH2* splicing events from RNA-seq analysis. (**D**) Relative expression levels of *ALKBH2* variants. Bar plots show the abundance of each variant using variant-specific primer pairs. *GAPDH* was used as the housekeeping gene for normalization. Data are expressed as mRNA copies per 10^6^ *GAPDH* copies. All qPCR experiments were performed in triplicate using independent biological replicates.

Nanopore sequencing confirmed the presence of the main *ALKBH2* mRNA (v.1: NM_001145374.2) and uncovered a new alternative transcript, *ALKBH2* v.6 (GenBank^®^ accession number: PX620490), which differentiates from the already annotated ones by the intron retention between exons 1 and 2 (Figure 2A).

**Figure 3:**
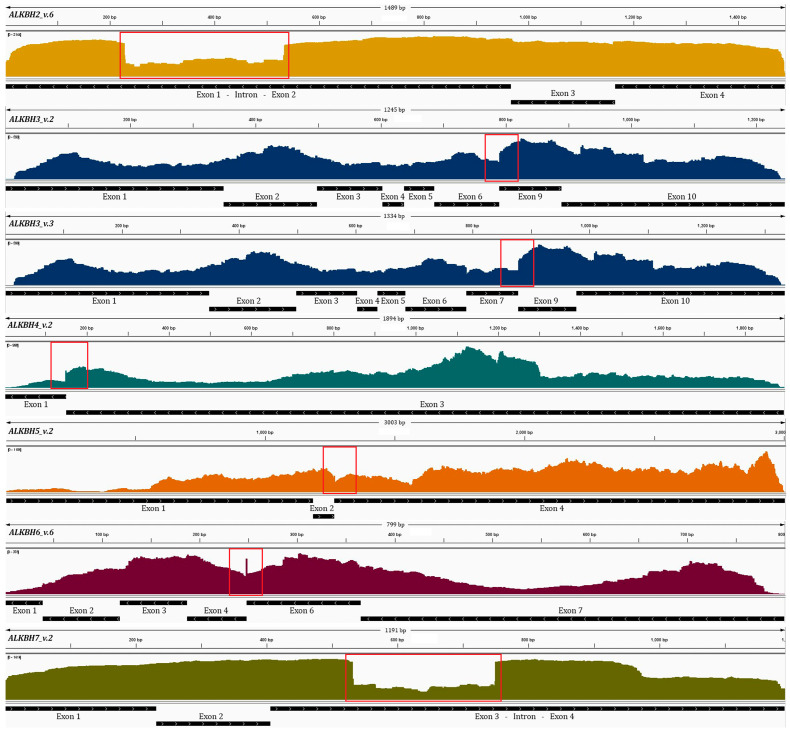
IGV visualization of the aligned sequencing reads corresponding to the investigated *ALKBH* genes, highlighting previously undescribed mRNAs that are characterized as coding transcripts, based on Poly-Ribo-Seq data from HEK-293T cells and TRAP-Seq from human pluripotent stem cells.

Bioinformatics analysis revealed that the translation initiation codon is located within exon 2, indicating that both isoforms share an identical open reading frame (ORF) encoding a 261-amino-acid protein. Thus, the retained intron affects only the 5′ untranslated region (5′ UTR) without altering the coding sequence. Short-read RNA sequencing supported the presence of the intron retention event, with reads spanning both junctions between the 3′ end of exon 1 and the retained intron, and between the 3′ end of the intron and the 5′ end of exon 2 (Figure 2B). Quantification of the MGI sequencing data revealed that the abundance of the retained intron is considerably lower than that of the canonical splice junctions (1/2, 2/3, and 3/4) defining the main transcript (Figure 2C). RT–qPCR analysis further demonstrated distinct expression patterns of ALKBH2 v.6 across cell lines. In all cancer-derived cell lines examined, ALKBH2 v.6 was significantly reduced compared to the canonical transcript (Figure 2D). Notably, statistical analysis revealed that the difference between v.1 and v.6 expression levels was non-significant (*p* > 0.05) only in non-cancerous MCF-10A cells. This suggests that both transcripts are expressed at comparable levels in this specific cell line, although further investigation is needed to fully elucidate the biological significance of this observation. Analysis of publicly available ribosome profiling datasets revealed ribosome occupancy along the 5′ UTRs of both ALKBH2 mRNAs, indicating active translation. These findings suggest that both transcripts produce the same protein, despite their structural differences in the 5′ UTR (Figure 3).

### 3.3. Three Novel ALKBH3 mRNAs (v.2–v.4) Are Generated by Multiple Exon Skipping Events

**Figure 4:**
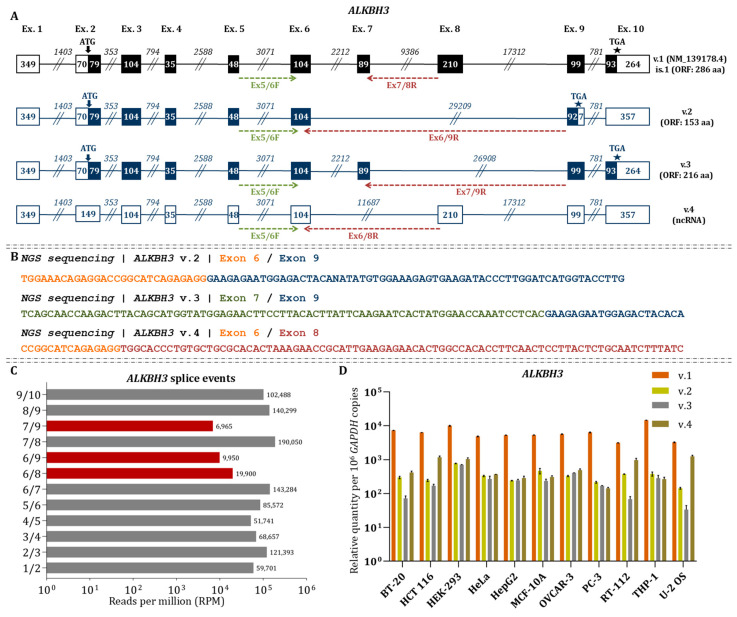
Identification and expression profiling of *ALKBH3* mRNAs (**A**) Schematic illustration of the *ALKBH3* transcript variants. Boxes represent exons, while introns are shown as lines. Black boxes indicate ORFs, while white boxes denote UTRs. Numbers within boxes and above lines correspond to the length of exons and introns, respectively. Arrows mark the start codon, and asterisks (★) indicate the stop codon. The transcript number and GenBank^®^ accession number are shown adjacent to each transcript. Green and red arrows denote the relative positions of the forward and reverse primers used for mRNA amplification. (**B**) Short read sequencing data confirming the newly detected splicing events that characterize the alternative *ALKBH3* mRNAs. (**C**) Comparison of RPM values for annotated and newly identified *ALKBH3* splicing events. (**D**) Relative expression levels of *ALKBH3* variants. Bar plots show the abundance of each variant using variant-specific primers. *GAPDH* was used as the housekeeping gene for normalization. Data are expressed as *ALKBH1* mRNA copies per 10^6^ *GAPDH* copies. All qPCR experiments were performed in triplicate using independent biological replicates.

*ALKBH3* v.1 (NM_139178.4) consists of exons 1–10 and has an ORF of 286 aa, with the start codon located in exon 2 and the stop codon in exon 10. Computational analysis revealed extensive exon-skipping events, giving rise to three previously unannotated mRNAs (*ALKBH3* v.2–v.4, GenBank^®^ accession number: PX620491–PX620493, accordingly) (Figure 4A). More specifically, *ALKBH3* v.2 is characterized by a novel splice junction between exons 6 and 9, skipping exons 7 and 8. This introduces a new stop codon in exon 9 but the transcript retains coding potential, producing a predicted protein of 153 aa. Furthermore, a splice junction between exons 7 and 9 generates the novel *ALKBH3* v.3 transcript. This newly described mRNA harbors the same start and stop codons with v.1 and encodes a predicted 216 aa protein. *ALKBH3* v.4 lacks exon 7, forming a splice junction between exons 6 and 8, and introduces a PTC, suggesting degradation via NMD or classification as a lncRNA (Figure 4A).

Short-read RNA sequencing confirmed the existence of novel splice junctions (6/9, 7/9, and 6/8), with reads spanning these positions (Figure 4B). In addition, quantitative analysis revealed that the newly observed events are less abundant compared to the annotated splice junctions, with the 7/9 junction (*ALKBH3* v.3) being the least frequent, followed by 6/9 (*ALKBH3* v.2). Among the novel events, the 6/8 junction (*ALKBH3* v.4) was the most common (Figure 4C). RT–qPCR quantification across multiple cell lines confirmed that all novel transcripts were expressed at lower levels than ALKBH3 v.1 (Figure 4D). Among these, *ALKBH3* v.2 generally showed higher expression than *ALKBH3* v.3, except from HepG2 and OVCAR-3 cells, where *ALKBH3* v.3 predominated. *ALKBH3* v.4 isoform was the most abundant among the novel transcripts in colorectal (HCT 116), urinary bladder (RT-112), and osteosarcoma (U-2 OS) cells, but the least abundant in prostate (PC-3) and monocytic leukemia (THP-1) cells (Figure 4D). Statistical comparisons between the alternative spliced variants confirmed that these differences in relative transcript abundance were significant (*p* < 0.05). Ribo-seq and TRAP-seq analyses revealed ribosome occupancy at the alternative splice junctions defining *ALKBH3* v.2 and v.3, indicating that both mRNAs are actively translated (Figure 3). These findings support the coding potential of both isoforms.

To assess amino acid conservation and structural variation, we aligned the predicted ALKBH3 protein sequences (v.1–v.3) using UniProt’s Clustal Omega “Align” function (Supplementary Data). The alignment revealed strong conservation across the N-terminal region, while the C-terminus diverged substantially in ALKBH3 v.2 due to the loss of exons 7 and 8, resulting in missing catalytic residues and truncation of the dioxygenase domain. Structural modeling predicted that both ALKBH3 v.2 and v.3 retain the N-terminal low-complexity region. Nonetheless, ALKBH3 v.2, (153 aa; 17.62 kDa) lacks the Fe(II) binding triad, active site, and α-ketoglutarate binding residues, encompassing only 64 of the 201 amino acids that form the catalytic core. In contrast, ALKBH3 v.3 (216 aa; 25.27 kDa) preserves much of the AlkB-like domain, including Thr133 (α-ketoglutarate site), His257 (Fe(II) site), and the C-terminal tail, but lacks His191, Asp193, Asp194, and Arg213 (Supplementary Figure S1). GO analysis revealed that the putative protein isoforms ALKBH3 v.2 and v.3 have distinct functional and biological profiles compared to the canonical protein. Regarding functionality, ALKBH3 v.2 exhibits increased scores for the activities of cytosine C-5 DNA demethylase and oxidative RNA demethylase, as well as for the catalytic activity acting on RNA, while its general catalytic activity score is reduced relative to v.1 and v.3. In addition, both isoforms v.2 and v.3 have lower prediction scores for mRNA N1-methyloadenosine dioxygenase activity, reflecting potential shifts in substrate specificity and biological roles (Supplementary Data).

### 3.4. Alternative Splicing Generates a Distinct ALKBH4 Splice Variant with Intron Retention

**Figure 5:**
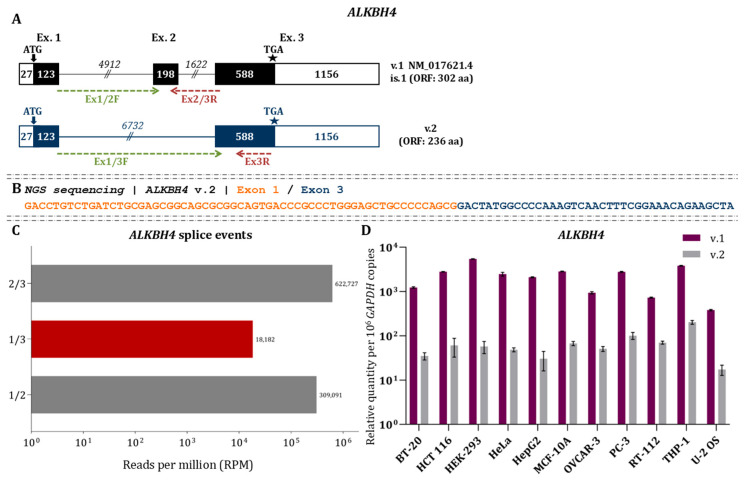
Structural organization and differential expression of *ALKBH4* mRNAs (**A**) Schematic representation of *ALKBH4* transcript variants. Exons are shown as boxes and introns as lines; black boxes denote coding regions and white boxes UTRs. Numbers indicate exon and intron lengths (nt). Arrows mark the start codon, and asterisks (★) the stop codon. Transcript numbers and GenBank^®^ accession IDs are indicated alongside each variant. Green and red arrows show the positions of the forward and reverse primers used for qPCR. (**B**) NGS data validating the detected splicing events of the *ALKBH4* mRNAs. (**C**) RPM values of the identified *ALKBH4* splicing events based on NGS data. (**D**) Relative expression of *ALKBH4* v.1 and v.2. Bar plots demonstrate variant abundance measured with variant-specific primers. Expression was normalized to *GAPDH* and is shown as mRNA copies per 10^6^ *GAPDH* copies.

*ALKBH4* v.1 (NM_017621.4) comprises three exons and encodes a 303-amino-acid protein, with the start codon located in exon 1 and the stop codon in exon 3. In addition, nanopore sequencing detected a previously uncharacterized mRNA (*ALKBH4* v.2, GenBank^®^ accession number: PX620494), characterized by a new splice junction connecting exons 1 and 3 (Figure 5A). Bioinformatics analysis indicated that *ALKBH4* v.2 retains the annotated initiation and stop codons, suggesting that it is protein-coding. Supporting this, both Ribo-seq and TRAP-seq analyses revealed ribosome occupancy at the alternative splice junction, indicating that *ALKBH4* v.2 is actively translated (Figure 3). Short-read RNA sequencing validated the existence of the novel splice junction between exons 1 and 3, with reads mapping precisely to this region (Figure 5B). Of note, quantification of the NGS data showed that this alternative event is rare compared to the canonical splice junctions 1/2 and 2/3 defining *ALKBH4* v.1 (Figure 5C). RT–qPCR analysis further confirmed that *ALKBH4* v.2 is significantly underrepresented relative to v.1 across all examined human cell lines, consistent with the RNA-seq results (Figure 5D).

Clustal Omega alignment of ALKBH4 v.1 and v.2 protein sequences revealed high overall conservation, with minor differences observed at the C-terminal end of the N-terminal low-complexity region and the start of the catalytic core (Supplementary Data). Structural modeling predicted that the putative ALKBH4 v.2 protein (236 aa; 26.05 kDa) retains the majority of the dioxygenase fold, including 41 of the 80 residues forming the N-terminal low-complexity region and 163 of the 190 residues of the catalytic core, as well as the C-terminal tail and all three residues of the Fe(II) binding triad (Supplementary Figure S2). The sequence-based GO analysis revealed that ALKBH4 v.2 exhibits an altered molecular function profile relative to ALKBH4 v.1, with certain GO terms showing higher or lower confidence scores. Notably, the variant lacks predicted catalytic activity on nucleic acids, whereas the enrichment scores for biological processes remain comparable between the two isoforms (Supplementary Data).

### 3.5. Exon Skipping Leads to ALKBH5 v.2, a Novel Splice Variant of the Major m6A Eraser

**Figure 6:**
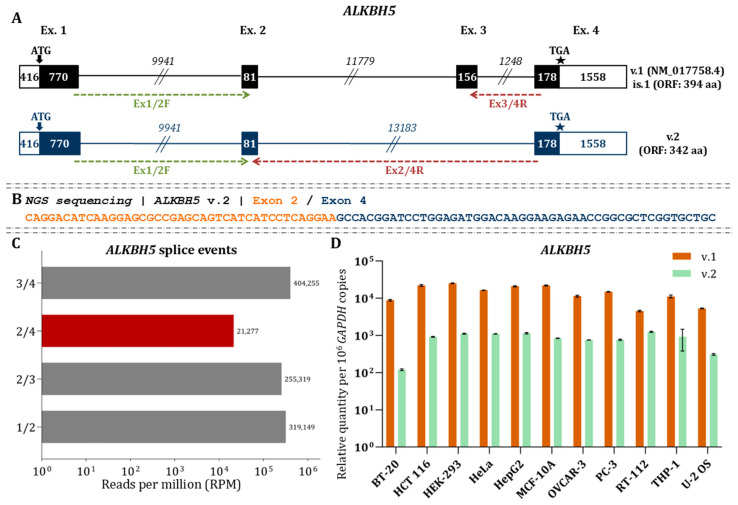
Analysis of *ALKBH5* transcripts (**A**) Black boxes represent coding exons, white boxes denote UTRs, and lines indicate introns. Numbers show exon and intron lengths (nt). Arrows mark the start codon, and asterisks (★) indicate the stop codon. GenBank^®^ accession IDs and ORF lengths are shown beside each variant. Green and red arrows indicate the transcript-specific forward and reverse primers used for qPCR. (**B**) NGS reads validating the detected splicing events of *ALKBH5* mRNAs. (**C**) RPM values of identified *ALKBH5* splicing events based on NGS data. (**D**) Relative expression of *ALKBH5* variants (v.1 and v.2). Bar plots show variant abundance measured with variant-specific primers. Expression was normalized to *GAPDH* and is presented as mRNA copies per 10^6^ *GAPDH* copies.

Direct RNA nanopore sequencing confirmed the presence of *ALKBH5* v.1 (NM_017758.4), which consists of four exons and encodes a 394-amino-acid protein. The start codon resides in exon 1 and the stop codon in exon 4. In addition to the annotated mRNA, we identified a novel transcript (*ALKBH5* v.2, GenBank^®^ accession number: PX620495) characterized by an alternative splice junction between exons 2 and 4 (Figure 6A).

ORF analysis revealed that both transcripts share the same initiation and termination codons, predicting a protein of 342 aa for *ALKBH5* v.2. NGS validated the existence of the novel junction between exons 2 and 4, with reads mapping precisely to this region (Figure 6B). Additionally, quantification of the RNA-seq data demonstrated that the abundance of the 2/4 junction is markedly lower compared to the annotated splice junctions (Figure 6C). RT-qPCR confirmed that *ALKBH5* v.2 is expressed in decreased levels compared to *ALKBH5* v.1 in all investigated cell lines (*p* < 0.05; Figure 6D). Supporting its potential coding capacity, ribosome profiling revealed ribosome occupancy at the splice junction defining *ALKBH5* v.2, indicating that this transcript is engaged in translation (Figure 3).

Clustal Omega alignment of ALKBH5 isoforms revealed high overall sequence conservation. The truncated v.2 isoform, which lacks the region encoded by exon 3, showed partial loss at the C-terminal segment corresponding mainly to charged residues and small or helix-disrupting residues (Supplementary Data). 3D structure of v.2 isoform (342 aa; 38.54 kDa) revealed that the protein retains key functional elements, including the N-terminal low-complexity region, 200 of the 208 residues forming the AlkB-like catalytic domain, substrate-binding loops, the Fe(II) binding triad, and the α-ketoglutarate binding site. It also preserves 41 of the 95 residues forming the C-terminal tail and 34 of 41 residues of the nuclear localization signal (NLS) cluster (Supplementary Figure S3). According to GO functional analysis, the putative protein isoform of ALKBH5 is predicted to exhibit similar activity with the main isoform. Both proteins displayed comparable molecular function prediction scores, with slightly higher values for v.1 in specific categories. The enrichment scores for biological processes were also largely similar, indicating overlapping functional potential (Supplementary Data).

### 3.6. Investigation of ALKBH6 Splice Junctions Reveals the Existence of ALKBH6 v.6

**Figure 7:**
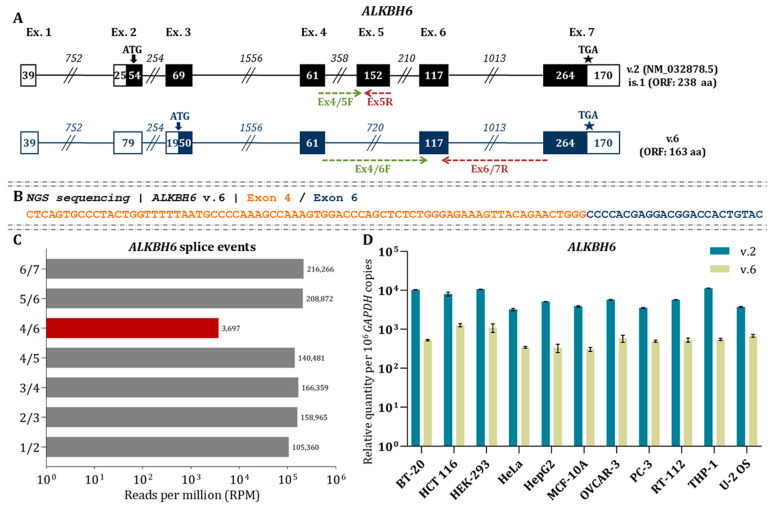
Detection and expression analysis of the *ALKBH6* variants (**A**) Coding exons are shown as black boxes, UTRs as white boxes, and introns as lines. Numbers indicate exon and intron lengths (nt). Arrows show the start codon, and asterisks (★) denote the stop codon. GenBank^®^ accession numbers and ORF lengths are indicated alongside each variant. Green and red arrows show the transcript-specific forward and reverse primers used for qPCR. (**B**) NGS reads confirming the detected splicing events of *ALKBH6* mRNAs. (**C**) RPM values of identified *ALKBH6* splicing events derived from NGS data. (**D**) Relative expression of *ALKBH6* variants. Bar plots display variant abundance measured with variant-specific primers. Expression was normalized to *GAPDH* and presented as mRNA copies per 10^6^ *GAPDH* copies.

Nanopore sequencing confirmed the presence of the main *ALKBH6* mRNA (v.2: NM_032878.5), which includes 7 exons and encodes a 238 aa protein, initiating at exon 2 and spanning to exon 7. A previously unreported splicing event between exons 4 and 6 was detected, generating a novel alternative transcript variant, *ALKBH6* v.6 (GenBank^®^ accession number: PX620496) (Figure 7A).

*ALKBH6* v.6 lacks the canonical start codon of *ALKBH6* v.1 but contains an ORF extending from exon 3, suggesting potential coding capacity. The presence of this novel splicing event detected by nanopore sequencing was validated by MGI sequencing experiments, where reads spanning the 3′ end of exon 4 and the 5′ end of exon 6 were detected (Figure 7B). Relative expression analysis showed that junction 4/6 is considerably less abundant than the canonical junctions based on RPM values (Figure 7C). Additionally, relative abundance of *ALKBH6* v.6 was found to be decreased compared to *ALKBH6* v.2 across all the investigated human cell lines (*p* < 0.05; Figure 7D). Ribo-seq and TRAP-seq of publicly available datasets revealed reads mapping to the 4/6 junction within actively translating ribosomes, supporting the potential translation of *ALKBH6* v.6 (Figure 3).

Clustal alignment between ALKBH6 v.2 and v.6 proteins revealed substantial differences in amino acid composition of the N-terminal region (Supplementary Data). Further analysis suggested that ALKBH6 v.6 (163 aa, 17.7 kDa) includes a truncated region in the catalytic core and the C-terminal tail. Moreover, His114 and Asp116 are conserved but occur at different positions relative to the canonical sequence, while N-terminal is completely different (Supplementary Figure S4). Consistent with partial domain loss, GO predictions indicate that ALKBH6 v.6 is likely catalytically inactive and participates in a limited set of biological processes (Supplementary Data).

### 3.7. Transcriptomics Analysis of ALKBH7 Reveals the Expression of the Novel Transcript ALKBH7 v.2

**Figure 8:**
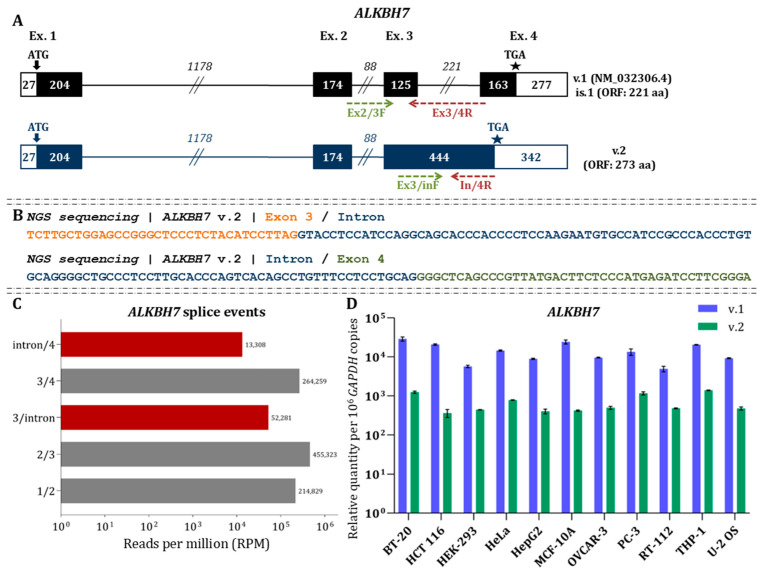
Identification and expression analysis of *ALKBH7* splice variants (**A**) Demonstration of *ALKBH7* transcripts. Coding exons are depicted as black boxes, UTRs as white boxes, and introns as connecting lines. Exon and intron lengths (nt) are indicated by numbers. Arrows mark the initiation codon, and asterisks (★) denote the termination codon. The GenBank^®^ accession numbers and ORF lengths are listed beside each variant. Green and red arrows indicate the positions of the forward and reverse primers used for variant-specific qPCR assays. (**B**) NGS read alignments supporting the identified *ALKBH7* splicing events. (**C**) RPM values representing the abundance of annotated and novel *ALKBH7* splicing events based on NGS data. (**D**) Relative expression of *ALKBH7* variants (v.1 and v.2). Bar plots display transcript abundance quantified using variant-specific primers. Expression values were normalized to *GAPDH* and are presented as mRNA copies per 10^6^ *GAPDH* copies.

*ALKBH7* v.1 (NM_032306.4) represents the only annotated protein-coding transcript. Its ORF initiates at exon 1 and ends at exon 4, encoding a 221 aa protein. dRNA-seq enabled the identification of a novel mRNA (*ALKBH7* v.2, GenBank^®^ accession number: PX620497), which is characterized by the retention of the intronic region between exons 3 and 4 (Figure 8A). As it preserves the annotated start codon, *ALKBH7* v.2 is predicted to be protein-coding, with an extended ORF of 273 aa. MGI datasets validated the intron retention event through reads spanning the 3′ end of exon 3 to the 5′ end of the retained intron, as well as reads covering the 3′ end of the intron to the 5′ end of exon 4 (Figure 8B).

Despite this, RPM analysis revealed that *ALKBH7* v.2 is expressed at lower levels than v.1, although its expression remains appreciable (Figure 8C). RT-qPCR further confirmed the lower abundance of *ALKBH7* v.2 compared to v.1 across tested cell lines (Figure 8D), a difference that was found to be statistically significant (*p* < 0.05). Analysis of Ribo-seq and TRAP-seq data unveiled sequencing reads mapped to the retained intronic region, thus supporting the translation of *ALKBH7* v.2 (Figure 3).

Clustal alignment of the ALKBH7 v.2 protein with the canonical isoform revealed that v.2 contains an extended C-terminal region, resulting in additional residues that are largely small, polar, or helix-disrupting (Supplementary Data). Structural analysis indicated that the N-terminal low-complexity region is conserved, while the catalytic dioxygenase core and the C-terminal tail of v.2 are entirely distinct from that of ALKBH7 v.1. Despite these differences, all residues essential for Fe(II) binding and α-ketoglutarate coordination are maintained, suggesting that the core catalytic function is likely preserved (Supplementary Figure S5). GO term analysis indicated limited molecular function for both isoforms. However, both are predicted to participate in multiple biological processes, with ALKBH7 v.2 showing a broader range of potential involvement (Supplementary Data).

## 4. Discussion

Eukaryotic cells exhibit a highly complex transcriptomic profile that directly influences the cellular proteome, with alternative splicing serving as the primary mechanism underlying this diversity [23,24,25]. Given that members of the *ALKBH* family encode RNA and DNA demethylases involved in epigenetic regulation, exploring their alternative splice variants offers valuable insights into how splicing may modulate their enzymatic activity, substrate specificity, and functional roles. Such investigation not only broadens our understanding of the way that *ALKBH* gene regulation affects the epigenetic landscape but may also elucidate their contribution to cancer development and progression. Comprehensive analysis of the sequencing data revealed multiple novel alternative transcripts arising from diverse splicing events, including exon skipping, intron retention, and exon truncation. Specifically, exon omission gave rise to *ALKBH3* v.2–v.4, *ALKBH4* v.1, *ALKBH5* v.2 and *ALKBH6* v.2, while *ALKBH2* v.6 and *ALKBH7* v.2 were characterized by intron retention. Interestingly, *ALKBH1* v.2 displayed a combination of exon skipping and truncation events.

The identification of these previously unannotated splice variants raises important questions regarding their functional significance relative to the canonical *ALKBH* transcripts. In silico ORF analysis indicated that most of the newly identified splice variants possess functional ORFs, suggesting that they are likely to be translated into functional protein isoforms. This implies that alternative splicing may further diversify the enzymatic repertoire of ALKBH demethylases, potentially modulating the methylation profile of cells. However, two novel transcripts (*ALKBH1* v.2, *ALKBH3* v.4) lack functional ORFs, inferring that they may function as long non-coding RNAs [26]. Given the growing relevance of non-coding RNAs originating from intronic and intergenic regions, these variants may play critical regulatory roles in cancer pathogenesis, potentially modulating the stability or translation of their canonical counterparts, rather than solely serving as substrates for NMD. The identification of intron retention in *ALKBH2* v.6 and *ALKBH7* v.2 further underscores the need to investigate how these specific intronic sequences may contribute to the epigenetic dysregulation observed in cancer. Moreover, emerging evidence highlights the critical role of lncRNAs in cancer pathogenesis, where they can act as molecular sponges for miRNAs, scaffolds for chromatin-modifying complexes, or decoys that modulate the stability and translation of their canonical counterparts [27]. Consequently, these non-coding *ALKBH* variants may contribute to the epigenetic dysregulation observed in malignancies, independent of protein translation. The distinction between coding and non-coding transcripts was based on the presence of a premature termination codon located >50–55 nucleotides upstream of the last exon–exon junction [28]. Such PTCs typically trigger NMD, leading to transcript degradation [29]. Thus, our results not only reveal a significant level of transcriptomic diversity within the *ALKBH* gene family but also highlight the complexity of ALKBH-mediated gene regulation, as the new variants exhibit both coding and non-coding potential.

Our analysis revealed that the newly detected splice variants exhibit distinct expression patterns. Compared to the canonical *ALKBH* mRNAs, most novel alternative transcripts were expressed at lower levels, except for *ALKBH2* v.6, whose expression was comparable to the canonical isoform in the non-cancerous MCF 10A and HEK-293 cell lines. In contrast, in cancerous cells, all novel splice variants were downregulated and differentially expressed, suggesting an altered regulation of the splicing machinery during tumorigenesis. The existence of distinct ALKBH expression profiles between non-cancerous and cancerous cells may contribute to the dysregulated methylation state characteristic of each cancer type. Given that methylation serves as a major epigenetic biomarker, the presence of alternative demethylase isoforms could be linked to genome instability, uncontrolled proliferation, and invasion of cancer cells, as well as activation of proto-oncogenes and transposons, and suppression of tumor suppressor genes [30,31]. Additionally, protein methylation has been shown to influence autophagy regulation [32], whereas RNA methylation modulates the tumor microenvironment and immune responses [33]. Interestingly, loss of DNA methylation has been associated with p53 and Rb-independent cellular senescence in cancer cells [34], highlighting the importance of further investigating the demethylation mechanisms in cancer biology.

To further evaluate the coding potential of the newly identified transcripts, we analyzed publicly available Ribo-seq and TRAP-seq datasets. The presence of ribosome-protected fragments mapped to several of these transcripts supports their active translation and strengthens the prediction that they may encode protein products. However, direct experimental evidence of protein expression and enzymatic activity remains to be established. Additional proteomic and biochemical validation will be required to confirm the translation and functional roles of these putative isoforms. Furthermore, while our in vitro models reveal distinct expression patterns, future studies utilizing primary clinical samples are essential to validate these findings and elucidate the specific mechanisms of action of ALKBH isoforms in the complex landscape of human malignancies.

Structural modeling of the predictive proteins yielded notable insights: certain isoforms, including ALKBH2 v.6 and ALKBH5 v.2, preserve most of the core structural motifs, such as the catalytic ALKBH core, the Fe(II)-binding triad, and the α-ketoglutarate binding site. This indicates that these isoforms are structurally compatible with enzymatic activity and share functional characteristics and subcellular localization with their canonical counterparts. However, several isoforms display partial or truncated structural conservation, suggesting altered or lost functionality. Such variants may modulate the activity of canonical isoforms through dominant-negative interactions or altered substrate specificity, localization, and regulatory dynamics. Notably, certain putative isoforms, such as the one encoded from *ALKBH6* v.6, exhibit substantial amino acid divergence and are predicted to be inactive, potentially serving as targets for degradation via the ubiquitin–proteasome system or lysosomal pathways [35].

Numerous studies have underscored the critical role of ALKBH demethylases in cellular homeostasis and epigenetic regulation. Alas, by influencing DNA, RNA and protein methylation states, they have been recognized for their role in cancer [8]. Additionally, ALKBH1 modulates translation efficiency by demethylating m1A lesions in tRNAs, thereby adjusting translational capacity in response to glucose availability, whereas the ncRNA *ALKBH1* v.2 may contribute to post-transcriptional regulation of its expression or interactions with RNA molecules [4]. On the contrary, ALKBH3-mediated m6A demethylation of tRNAs enhances translational efficiency in tumor cells [36], illustrating that methylation at different nucleotide positions can exert opposing effects, while the presence of alternative *ALKBH3* mRNAs may further modulate or fine-tune its enzymatic activity and functional impact. Similarly, *ALKBH4* has been found to enhance protein synthesis via uridine modification in tRNA, while the newly identified *ALKBH4* v.2 may regulate its role in translation [37]. Furthermore, mitochondrial ALKBH7 demethylates m2,2G and m1A positions in pre-tRNAs for Ile and Leu1 within polycistronic mitochondrial RNAs, thereby promoting tRNA maturation and mitochondrial translation [38]. Given the pivotal role of mitochondria in cancer pathophysiology, the emergence of alternative *ALKBH7* transcripts could influence mitochondrial function and metabolic adaptation in cancer cells.

Overall, our study expands the current understanding of the *ALKBH* gene family by uncovering a previously uncharacterized spectrum of alternative splice events. The transition from in vitro identification to mechanistic characterization in clinical tissues remains essential. While our findings reveal distinct expression patterns in cell lines, future studies utilizing primary patient samples are required to validate these findings in a clinical context. As methylation dynamics are fundamental to gene expression, differentiation, and development, isoform divergence may have profound implications for cellular homeostasis and disease.

## 5. Conclusions

In this study, we aimed to elucidate the transcriptomic landscape of the *ALKBH* gene family. By integrating both short-read and long-read sequencing technologies, we systematically characterized alternative splicing events among the nine Fe^2+^/α-ketoglutarate-dependent ALKBH dioxygenases, which play essential roles in regulating the cellular methylation state through the demethylation of nucleic acids and proteins. Comprehensive analysis of the sequencing data revealed multiple previously uncharacterized *ALKBH* alternative transcripts arising from diverse splicing events. Furthermore, the distinct expression patterns of *ALKBH* mRNAs observed between malignant and non-malignant cells highlight their potential as biomarkers or therapeutic targets. Continued exploration of ALKBH isoforms at the proteomic and functional levels may thus pave the way for novel diagnostic and therapeutic strategies in cancer biology.

## Data Availability

The nucleotide sequences of the novel mRNA transcripts described in the present study have been deposited to the GenBank^®^ database (https://www.ncbi.nlm.nih.gov/genbank/) and correspond to the accession numbers PX620489—PX620497.

## Supplementary Materials

The following supporting information can be downloaded at: https://www.mdpi.com/article/10.3390/cimb48030251/s1, Figure S1: Protein models of the described putative ALKBH3 isoforms generated using AlphaFold3; Figure S2: Structural demonstration of the ALKBH4 proteins obtained by AlphaFold3 software; Figure S3: Predicted 3D structure models of the ALKBH5 proteins.; Figure S4: AlphaFold3-generated models illustrate the structural organization of ALKBH6 isoforms; Figure S5: Predicted 3D structure models of ALKBH7 isoforms.; Table S1: Panel of cell lines used in this study and their corresponding tissue of origin; Table S2: List of primers that were used for the specific qPCR amplification of mRNAs of the *ALKBH* gene family.

## Abbreviations

The following abbreviations are used in this manuscript:

5′ UTR	5′ untranslated region
aa	Amino acid
ALKBH	AlkB Homolog
ATCC	American Type Culture Collection
cDNA	Complementary DNA
DNBSEQ	DNA Nanoball Sequencing
dRNA-seq	Direct RNA sequencing
ExPASy	Expert Protein Analysis System
FTO	Fat Mass and Obesity-Associated Protein
GAPDH	Glyceraldehyde-3-phosphate dehydrogenase
GO	Gene Ontology
HISAT2	Hierarchical Indexing for Spliced Alignment of Transcripts 2
IGV	Integrative Genomics Viewer
kDa	Kilodalton
lncRNAs	Long non-coding RNA
m1A	N1-methyladenosine
m2,2G	N2,N2-dimethylguanosine
m3C	N3-methylcytidine
m6A	N6-methyladenosine
mRNA	Messenger RNA
NCBI	National Center for Biotechnology Information
ncRNA	Non-coding RNA
NGS	Next-Generation Sequencing
NLS	Nuclear localization signal
NMD	Nonsense-mediated decay
ONT	Oxford Nanopore Technologies
ORF	Open Reading Frame
PTC	Premature termination codon
qPCR	Quantitative polymerase chain reaction
Rb	Retinoblastoma
RMX	RNA Adapter Mix
RPM	Reads Per Million
RT	Reverse Transcription
RTA	RT Adapter
SD	Standard deviation
SRA	Sequence Read Archive
TNBC	Triple-Negative Breast Cancer
TRAP-Seq	Translating Ribosome Affinity Purification Sequencing
tRNA	transfer RNA
