# Supplementary material for: Identification of Novel Alternative Transcripts of the Human ALKBH Gene Family and Investigation of Their Unique Expression Signatures in Cancer Cells
Source: Curr Issues Mol Biol. 2026 Feb 26;48(3):251. doi: 10.3390/cimb48030251 (PMC13026028; doi:10.3390/cimb48030251)
Supplement: Supplementary file 1 [file cimb-48-00251-s001.zip › supplementary data.pdf]

Protein sequence alignment

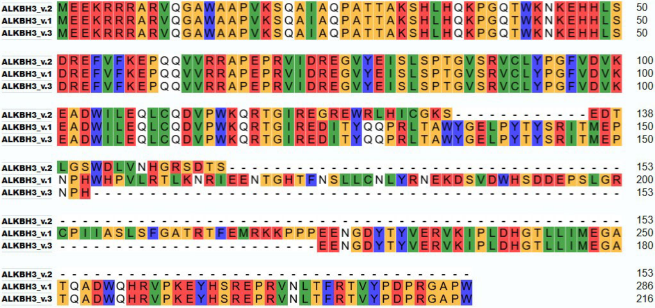
ALKBH3_v.2 MEEKRRRARVQGAWAAPVKSQAIAQPATTAKSHLHQKPGQTWKNKEHHLS 50
ALKBH3_v.1 MEEKRRRARVQGAWAAPVKSQAIAQPATTAKSHLHQKPGQTWKNKEHHLS 50
ALKBH3_v.3 MEEKRRRARVQGAWAAPVKSQAIAQPATTAKSHLHQKPGQTWKNKEHHLS 50
ALKBH3_v.2 DREFVFKEPQQVVRRAPEPRVIDREGVYEISLSPTGVSRVCLYPGFVDVK 100
ALKBH3_v.1 DREFVFKEPQQVVRRAPEPRVIDREGVYEISLSPTGVSRVCLYPGFVDVK 100
ALKBH3_v.3 DREFVFKEPQQVVRRAPEPRVIDREGVYEISLSPTGVSRVCLYPGFVDVK 100
ALKBH3_v.2 EADWILEQLCQDVPWKQRTGIREGREWRLHICGKS------------EDT 138
ALKBH3_v.1 EADWILEQLCQDVPWKQRTGIREDITYQQPRLTAWYGELPYTYSRITMEP 150
ALKBH3_v.3 EADWILEQLCQDVPWKQRTGIREDITYQQPRLTAWYGELPYTYSRITMEP 150
ALKBH3_v.2 LGSWDLVNHGRSDTS----------------------------------- 153
ALKBH3_v.1 NPHWHPVLRTLKNRIEENTGHTFNSLLCNLYRNEKDSVDWHSDDEPSLGR 200
ALKBH3_v.3 NPH--- 153
ALKBH3_v.2 -- 153
ALKBH3_v.1 CPIIASLSFGATRTFEMRKKPPPEENGDYTYVERVKIPLDHGTLLIMEGA 250
ALKBH3_v.3 -----------------------EENGDYTYVERVKIPLDHGTLLIMEGA 180
ALKBH3_v.2 ------------------------------------ 153
ALKBH3_v.1 TQADWQHRVPKEYHSREPRVNLTFRTVYPDPRGAPW 286
ALKBH3_v.3 TQADWQHRVPKEYHSREPRVNLTFRTVYPDPRGAPW 216

```
ALKBH4_v.1  MAAAAAETPEVLRECGCKGIRTCLICERQRGSDPPWELPPAKTYRFIYCS   50
ALKBH4_v.2  MAAAAAETPEVLRECGCKGIRTCLICERQRGSDPPWELPPA---------   41

ALKBH4_v.1  DTGWAVGTEESDFEGWAFPFPGVMLIEDFVTREEEAELVRLMDRDPWKLS  100
ALKBH4_v.2  --------------------------------------------------   41

ALKBH4_v.1  QSGRRKQDYGPKVNFRKQKLKTEGFCGLPSFSREVVRRMGLYPGLEGFRP  150
ALKBH4_v.2  -------DYGPKVNFRKQKLKTEGFCGLPSFSREVVRRMGLYPGLEGFRP   84

ALKBH4_v.1  VEQCNLDYCPERGSAIDPHLDDAWLWGERLVSLNLLSPTVLSMCREAPGS  200
ALKBH4_v.2  VEQCNLDYCPERGSAIDPHLDDAWLWGERLVSLNLLSPTVLSMCREAPGS  134

ALKBH4_v.1  LLLCSAPSAAPEALVDSVIAPSRSVLCQEVEVAIPLPARSLLVLTGAARH  250
ALKBH4_v.2  LLLCSAPSAAPEALVDSVIAPSRSVLCQEVEVAIPLPARSLLVLTGAARH  184

ALKBH4_v.1  QWKHAIHRRHIEARRVCVTFRELSAEFGPGGRQQELGQELLRIALSFQGR  300
ALKBH4_v.2  QWKHAIHRRHIEARRVCVTFRELSAEFGPGGRQQELGQELLRIALSFQGR  234

ALKBH4_v.1  PV                                                 302
ALKBH4_v.2  PV                                                 236
```

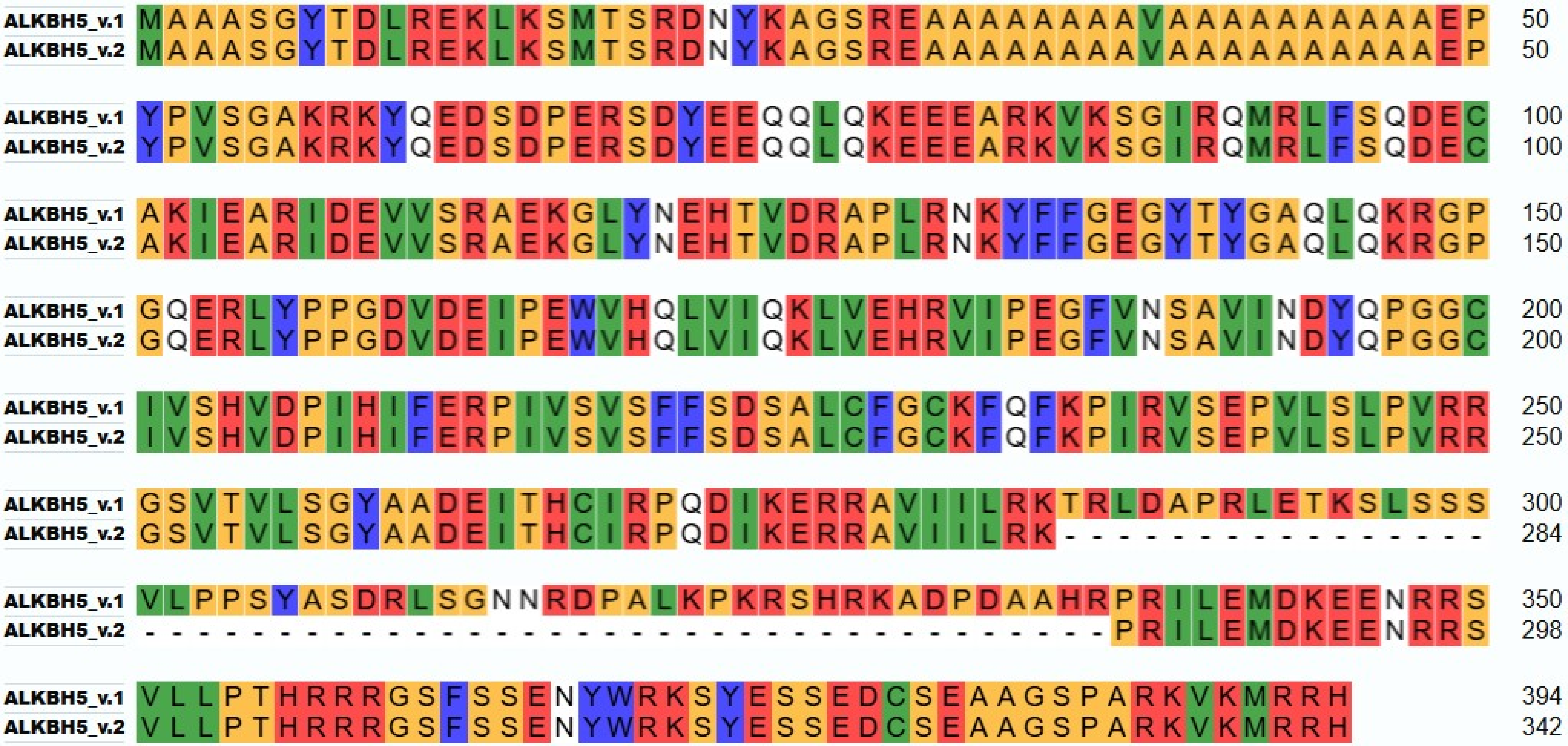
ALKBH5_v.1 MAAASGYTDLREKLKSMTSRDNYKAGSREAAAAAAAAVAAAAAAAAAAEP 50
ALKBH5_v.2 MAAASGYTDLREKLKSMTSRDNYKAGSREAAAAAAAAVAAAAAAAAAAEP 50
ALKBH5_v.1 YPVSGAKRKYQEDSDPERSDYEEQQLQKEEEARKVKSGIRQMRLFSQDEC 100
ALKBH5_v.2 YPVSGAKRKYQEDSDPERSDYEEQQLQKEEEARKVKSGIRQMRLFSQDEC 100
ALKBH5_v.1 AKIEARIDEVVSRAEKGLYNEHTVDRAPLRNKYFFGEGYTYGAQLQKRGP 150
ALKBH5_v.2 AKIEARIDEVVSRAEKGLYNEHTVDRAPLRNKYFFGEGYTYGAQLQKRGP 150
ALKBH5_v.1 GQERLYPPGDVDEIPEWVHQLVIQKLVEHRVIPEGFVNSAVINDYQPGGC 200
ALKBH5_v.2 GQERLYPPGDVDEIPEWVHQLVIQKLVEHRVIPEGFVNSAVINDYQPGGC 200
ALKBH5_v.1 IVSHVDPIHIFERPIVSVSFFSDSALCFGCKFQFKPIRVSEPVLSLPVRR 250
ALKBH5_v.2 IVSHVDPIHIFERPIVSVSFFSDSALCFGCKFQFKPIRVSEPVLSLPVRR 250
ALKBH5_v.1 GSVTVLSGYAADEITHCIRPQDIKERRAVIILRKTRLDAPRLETKSLSSS 300
ALKBH5_v.2 GSVTVLSGYAADEITHCIRPQDIKERRAVIILRK---------------- 284
ALKBH5_v.1 VLPPSYASDRLSGNNRDPALKPKRSHRKADPDAAHRPRILEMDKEENRRS 350
ALKBH5_v.2 ------------------------------------PRILEMDKEENRRS 298
ALKBH5_v.1 VLLPTHRRRGSFSSENYWRKSYESSEDCSEAAGSPARKVKMRRH 394
ALKBH5_v.2 VLLPTHRRRGSFSSENYWRKSYESSEDCSEAAGSPARKVKMRRH 342

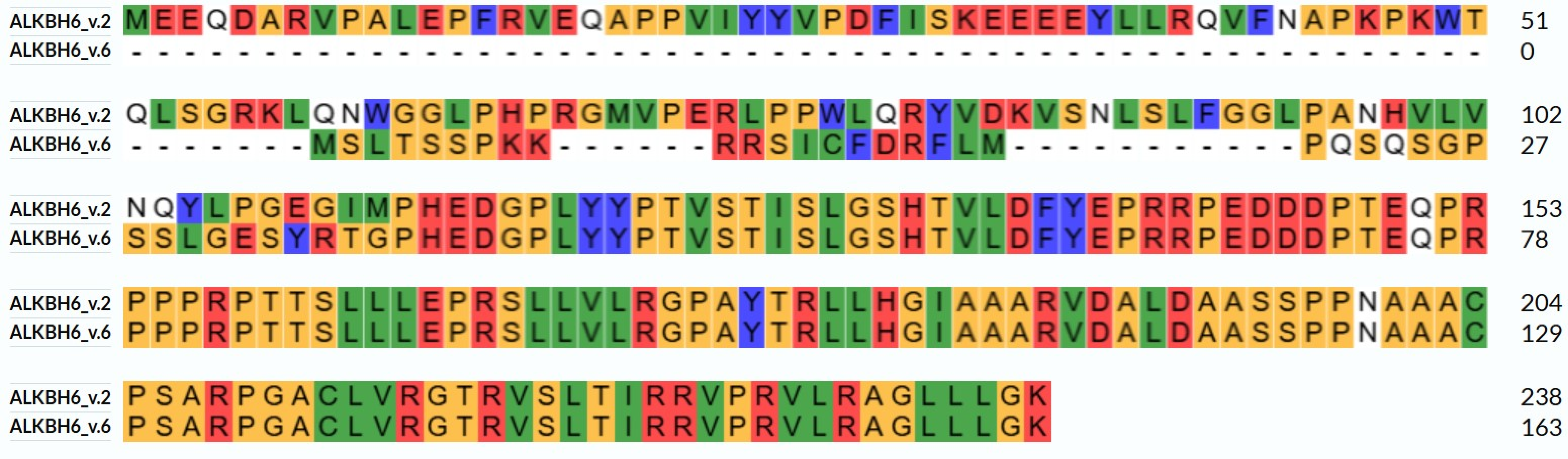

ALKBH6_v.2 MEEQDARVPALEPFRVEQAPPVIYYVPDFISKEEEEYLLRQVFNAPKPKWT 51
ALKBH6_v.6 --- 0
ALKBH6_v.2 QLSGRKLQNWGGLPHPRGMVPERLPPWLQRYVDKVSNLSLFGGLPANHVLV 102
ALKBH6_v.6 -------MSLTSSPKK------RRSICFDRFLM-----------PQSQSGP 27
ALKBH6_v.2 NQYLPGEGIMPHEDGPLYYPTVSTISLGSHTVLDFYEPRRPEDDDPTEQPR 153
ALKBH6_v.6 SSLGESYRTGPHEDGPLYYPTVSTISLGSHTVLDFYEPRRPEDDDPTEQPR 78
ALKBH6_v.2 PPPRPTTSLLLEPRSLLVLRGPAYTRLLHGIAAARVDALDAASSPPNAAAC 204
ALKBH6_v.6 PPPRPTTSLLLEPRSLLVLRGPAYTRLLHGIAAARVDALDAASSPPNAAAC 129
ALKBH6_v.2 PSARPGACLVRGTRVSLTIRRVPRVLRAGLLLGK 238
ALKBH6_v.6 PSARPGACLVRGTRVSLTIRRVPRVLRAGLLLGK 163

```
ALKBH7_v.1  MAGTGLLALRTLPGPSWVRGSGPSVLSRLQDAAVVRPGFLSTAEEETLSR  50
ALKBH7_v.2  MAGTGLLALRTLPGPSWVRGSGPSVLSRLQDAAVVRPGFLSTAEEETLSR  50

ALKBH7_v.1  ELEPELRRRRYEYDHWDAAIHGFRETEKSRWSEASRAILQRVQAAAFGPG  100
ALKBH7_v.2  ELEPELRRRRYEYDHWDAAIHGFRETEKSRWSEASRAILQRVQAAAFGPG  100

ALKBH7_v.1  QTLLSSVHVLDLEARGYIKPHVDSIKFCGATIAGLSLLSPSVMRLVHTQE  150
ALKBH7_v.2  QTLLSSVHVLDLEARGYIKPHVDSIKFCGATIAGLSLLSPSVMRLVHTQE  150

ALKBH7_v.1  PGEWLELLLEPGSLYILRGSARYDFSH--EILRDE--------ESFFGER  190
ALKBH7_v.2  PGEWLELLLEPGSLYILRYLHPGSTHPSKNVPSAHPVPWVLSLNPALPED  200

ALKBH7_v.1  RIPRGRRISVICRSLPEGMGPGESGQPPPAC-------------------  221
ALKBH7_v.2  AFTPGSVWEAAGGWNPGGWNPGLPGAGAALLAPSHSLFLPAGAQPVMTSP  250

ALKBH7_v.1  -----------------------  221
ALKBH7_v.2  MRSFGMKSPSLGNAGFPGAGASP  273
```

Gene Ontology Enrichment Analysis

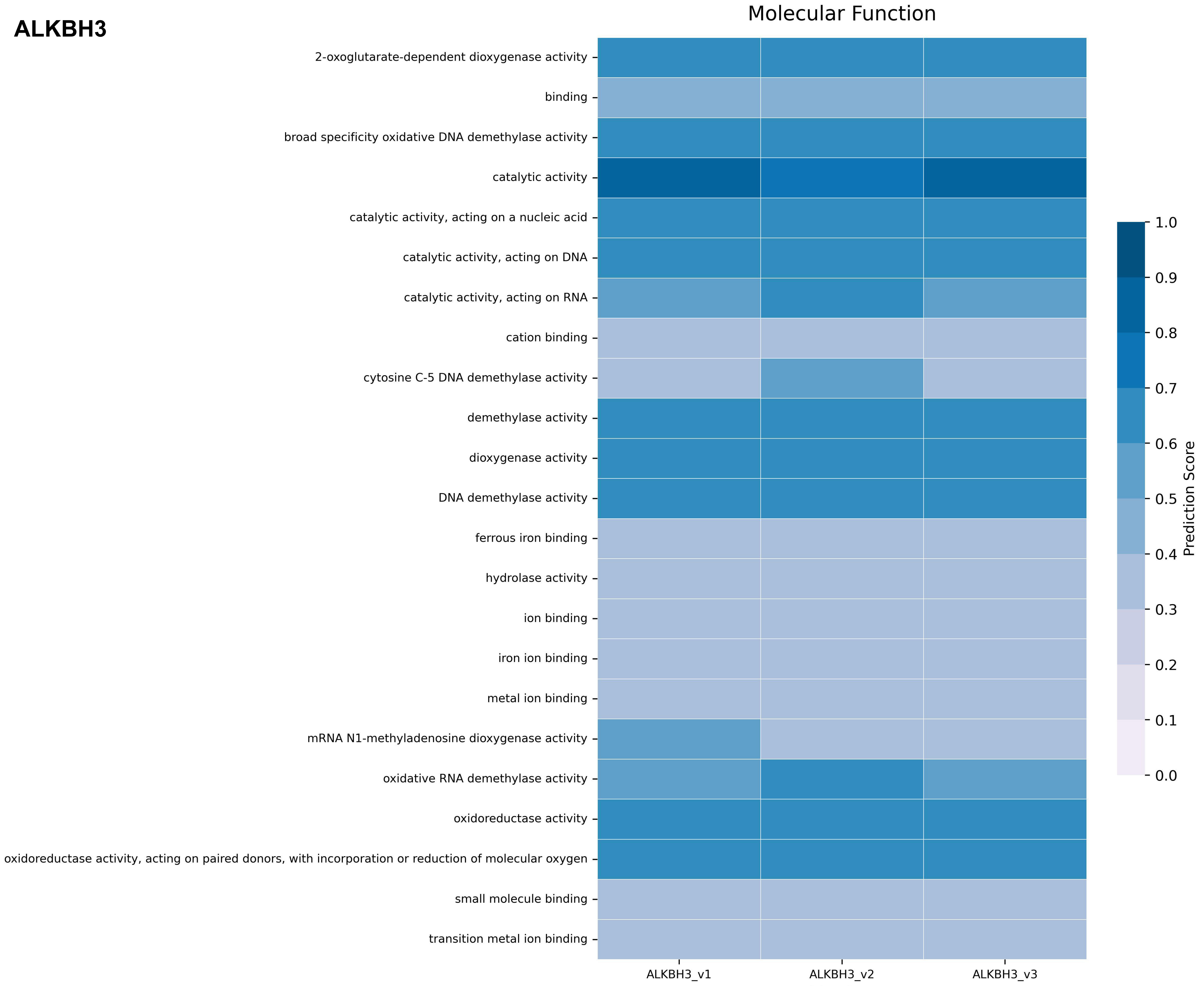

ALKBH3
Molecular Function
2-oxoglutarate-dependent dioxygenase activity
binding
broad specificity oxidative DNA demethylase activity
catalytic activity
catalytic activity, acting on a nucleic acid
catalytic activity, acting on DNA
catalytic activity, acting on RNA
cation binding
cytosine C-5 DNA demethylase activity
demethylase activity
dioxygenase activity
DNA demethylase activity
ferrous iron binding
hydrolase activity
ion binding
iron ion binding
metal ion binding
mRNA N1-methyladenosine dioxygenase activity
oxidative RNA demethylase activity
oxidoreductase activity
oxidoreductase activity, acting on paired donors, with incorporation or reduction of molecular oxygen
small molecule binding
transition metal ion binding
ALKBH3_v1
ALKBH3_v2
ALKBH3_v3
Prediction Score
0.0
0.1
0.2
0.3
0.4
0.5
0.6
0.7
0.8
0.9
1.0

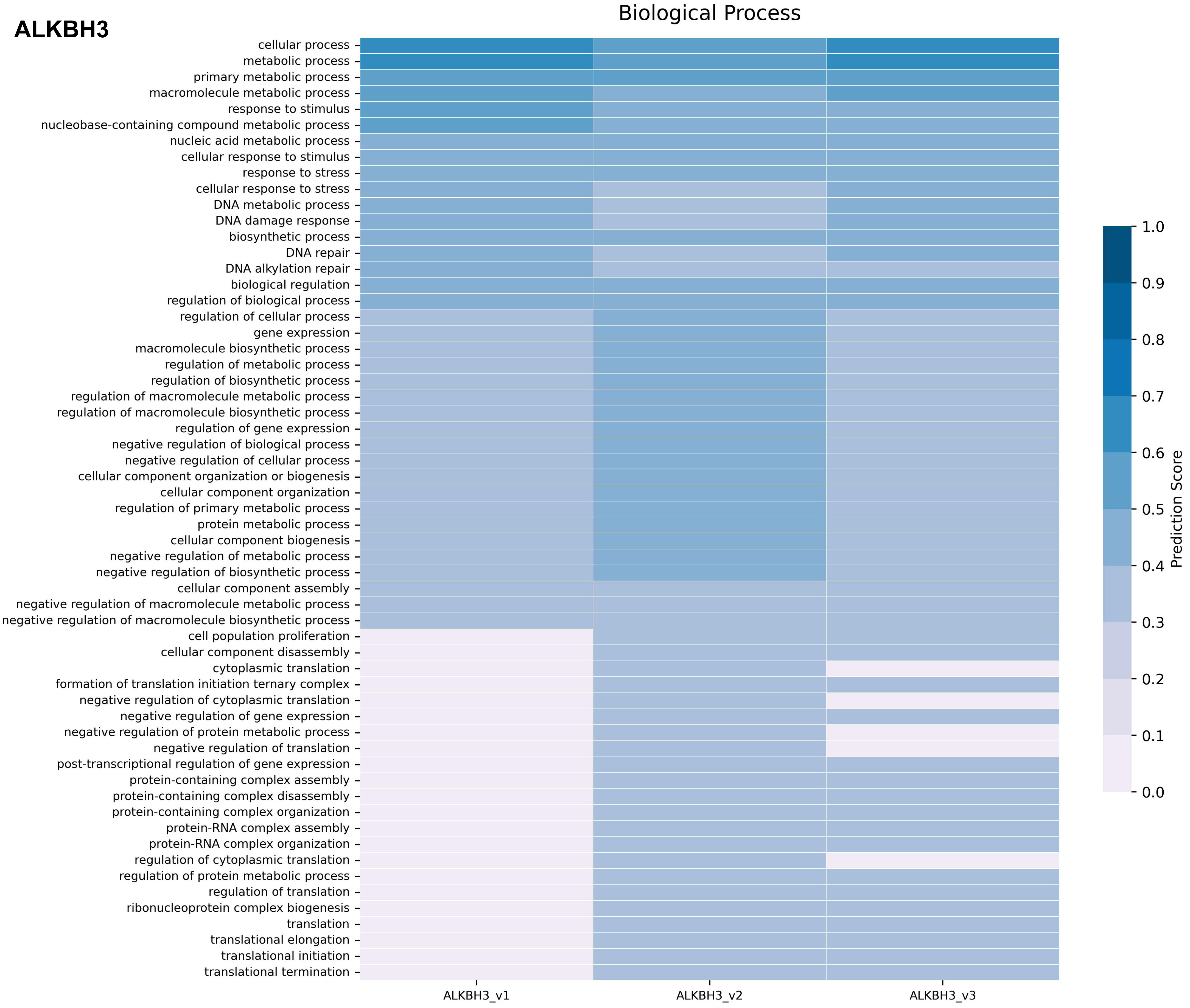
ALKBH3
Biological Process
cellular process
metabolic process
primary metabolic process
macromolecule metabolic process
response to stimulus
nucleobase-containing compound metabolic process
nucleic acid metabolic process
cellular response to stimulus
response to stress
cellular response to stress
DNA metabolic process
DNA damage response
biosynthetic process
DNA repair
DNA alkylation repair
biological regulation
regulation of biological process
regulation of cellular process
gene expression
macromolecule biosynthetic process
regulation of metabolic process
regulation of biosynthetic process
regulation of macromolecule metabolic process
regulation of macromolecule biosynthetic process
regulation of gene expression
negative regulation of biological process
negative regulation of cellular process
cellular component organization or biogenesis
cellular component organization
regulation of primary metabolic process
protein metabolic process
cellular component biogenesis
negative regulation of metabolic process
negative regulation of biosynthetic process
cellular component assembly
negative regulation of macromolecule metabolic process
negative regulation of macromolecule biosynthetic process
cell population proliferation
cellular component disassembly
cytoplasmic translation
formation of translation initiation ternary complex
negative regulation of cytoplasmic translation
negative regulation of gene expression
negative regulation of protein metabolic process
negative regulation of translation
post-transcriptional regulation of gene expression
protein-containing complex assembly
protein-containing complex disassembly
protein-containing complex organization
protein-RNA complex assembly
protein-RNA complex organization
regulation of cytoplasmic translation
regulation of protein metabolic process
regulation of translation
ribonucleoprotein complex biogenesis
translation
translational elongation
translational initiation
translational termination
ALKBH3_v1
ALKBH3_v2
ALKBH3_v3
Prediction Score
1.0
0.9
0.8
0.7
0.6
0.5
0.4
0.3
0.2
0.1
0.0

ALKBH4

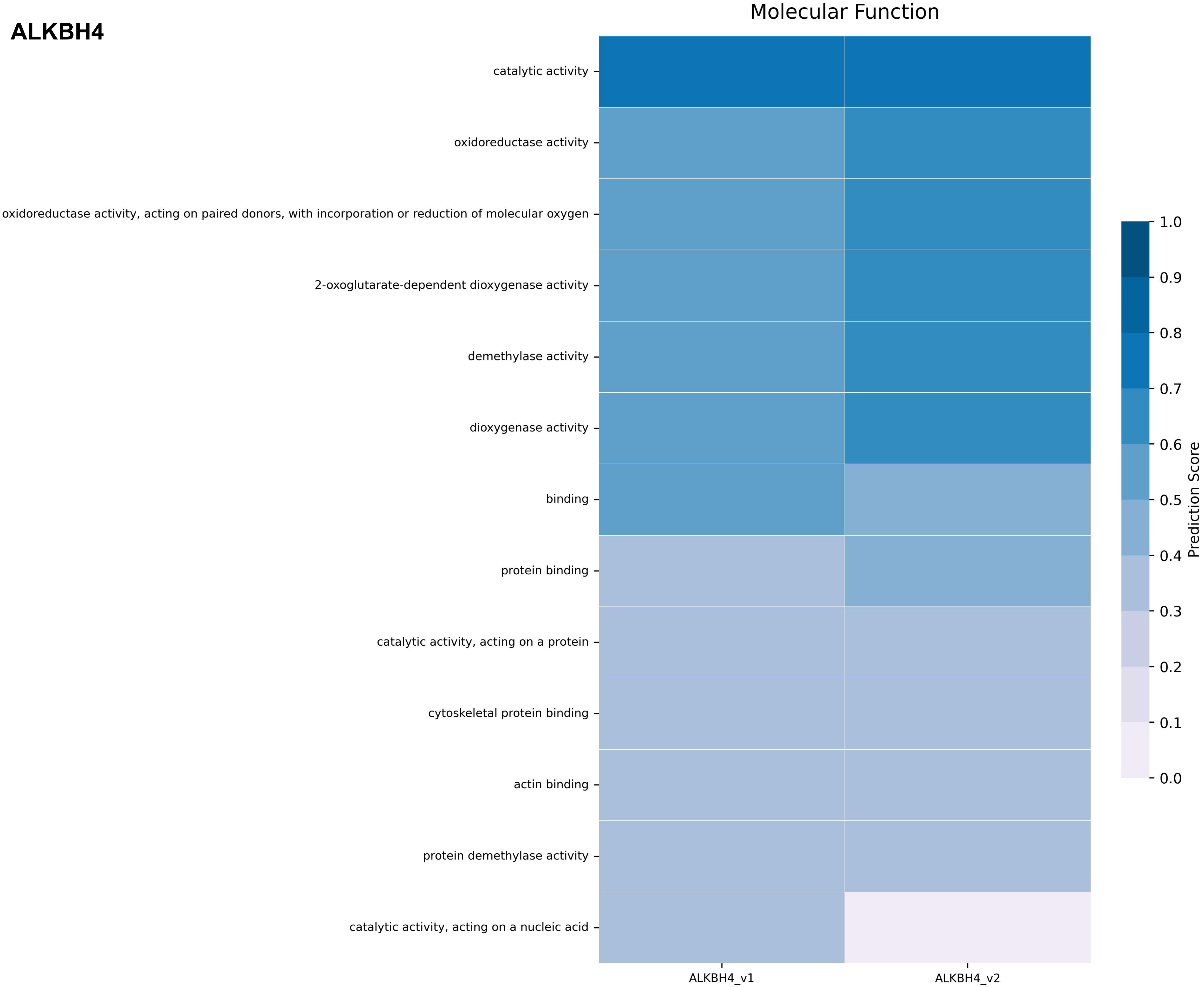

Molecular Function
catalytic activity
oxidoreductase activity
oxidoreductase activity, acting on paired donors, with incorporation or reduction of molecular oxygen
2-oxoglutarate-dependent dioxygenase activity
demethylase activity
dioxygenase activity
binding
protein binding
catalytic activity, acting on a protein
cytoskeletal protein binding
actin binding
protein demethylase activity
catalytic activity, acting on a nucleic acid
ALKBH4_v1
ALKBH4_v2
Prediction Score
1.0
0.9
0.8
0.7
0.6
0.5
0.4
0.3
0.2
0.1
0.0

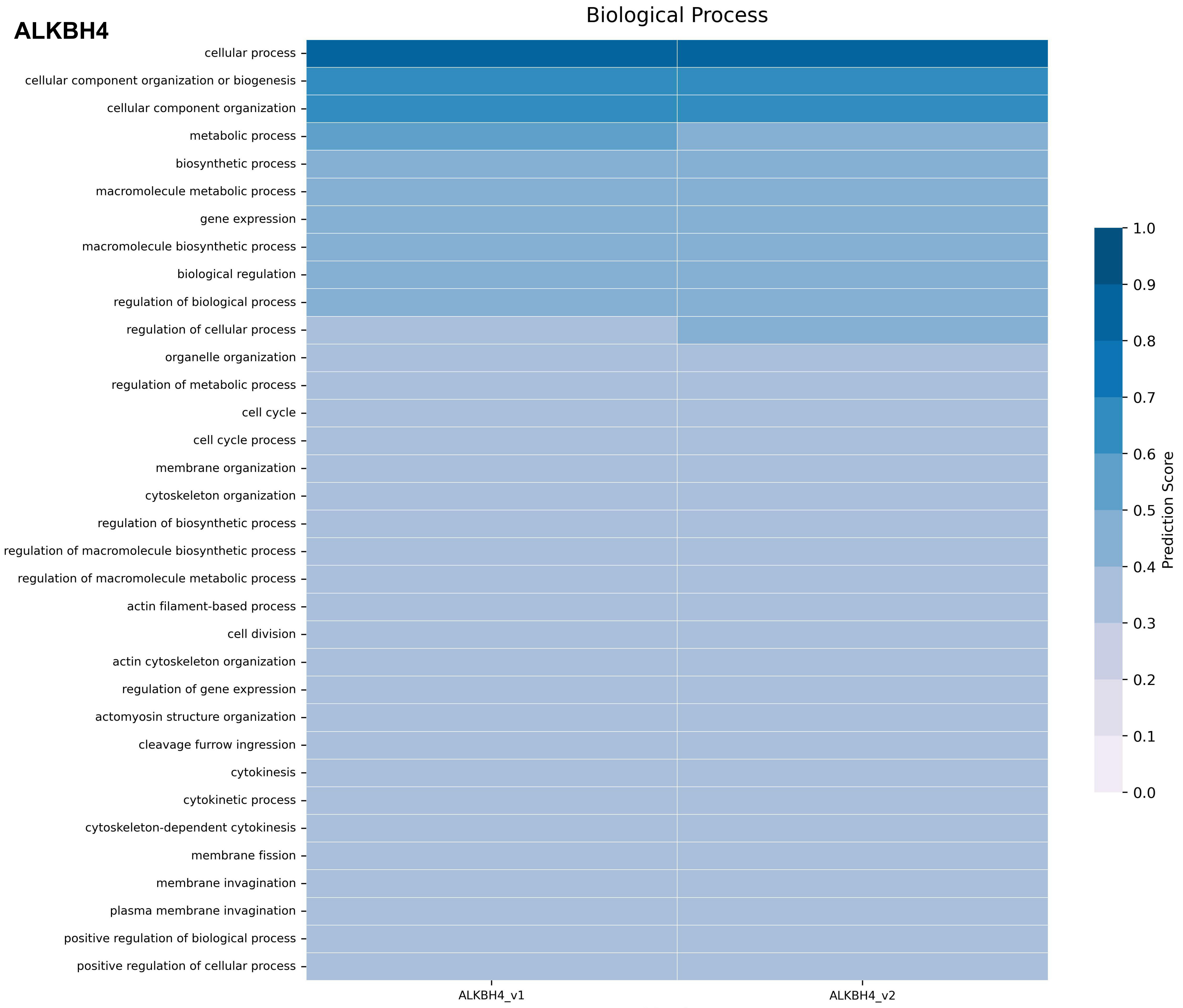
ALKBH4
Biological Process
cellular process
cellular component organization or biogenesis
cellular component organization
metabolic process
biosynthetic process
macromolecule metabolic process
gene expression
macromolecule biosynthetic process
biological regulation
regulation of biological process
regulation of cellular process
organelle organization
regulation of metabolic process
cell cycle
cell cycle process
membrane organization
cytoskeleton organization
regulation of biosynthetic process
regulation of macromolecule biosynthetic process
regulation of macromolecule metabolic process
actin filament-based process
cell division
actin cytoskeleton organization
regulation of gene expression
actomyosin structure organization
cleavage furrow ingression
cytokinesis
cytokinetic process
cytoskeleton-dependent cytokinesis
membrane fission
membrane invagination
plasma membrane invagination
positive regulation of biological process
positive regulation of cellular process
ALKBH4_v1
ALKBH4_v2
Prediction Score
0.0
0.1
0.2
0.3
0.4
0.5
0.6
0.7
0.8
0.9
1.0

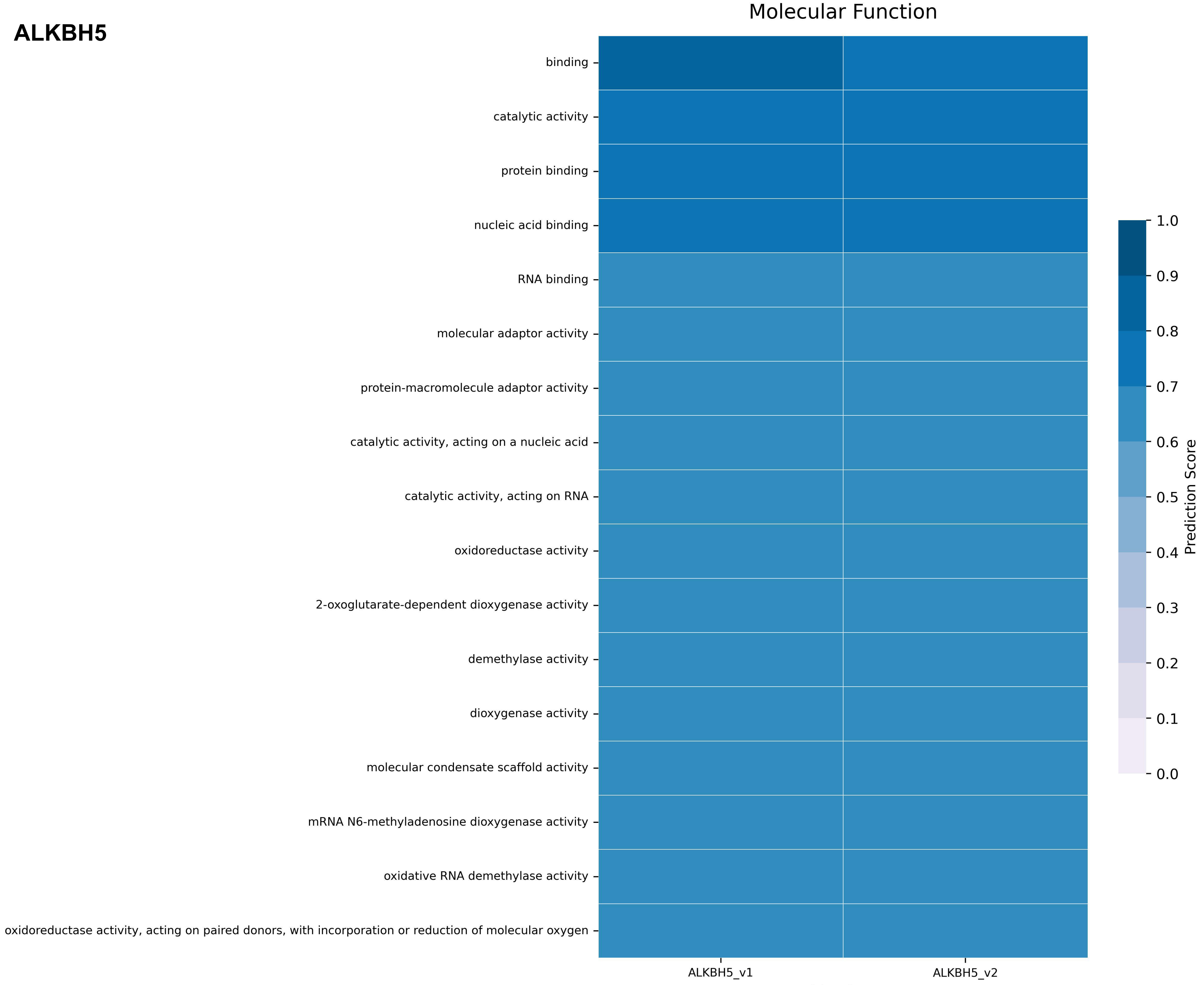
ALKBH5
Molecular Function
binding
catalytic activity
protein binding
nucleic acid binding
RNA binding
molecular adaptor activity
protein-macromolecule adaptor activity
catalytic activity, acting on a nucleic acid
catalytic activity, acting on RNA
oxidoreductase activity
2-oxoglutarate-dependent dioxygenase activity
demethylase activity
dioxygenase activity
molecular condensate scaffold activity
mRNA N6-methyladenosine dioxygenase activity
oxidative RNA demethylase activity
oxidoreductase activity, acting on paired donors, with incorporation or reduction of molecular oxygen
ALKBH5_v1
ALKBH5_v2
Prediction Score
1.0
0.9
0.8
0.7
0.6
0.5
0.4
0.3
0.2
0.1
0.0

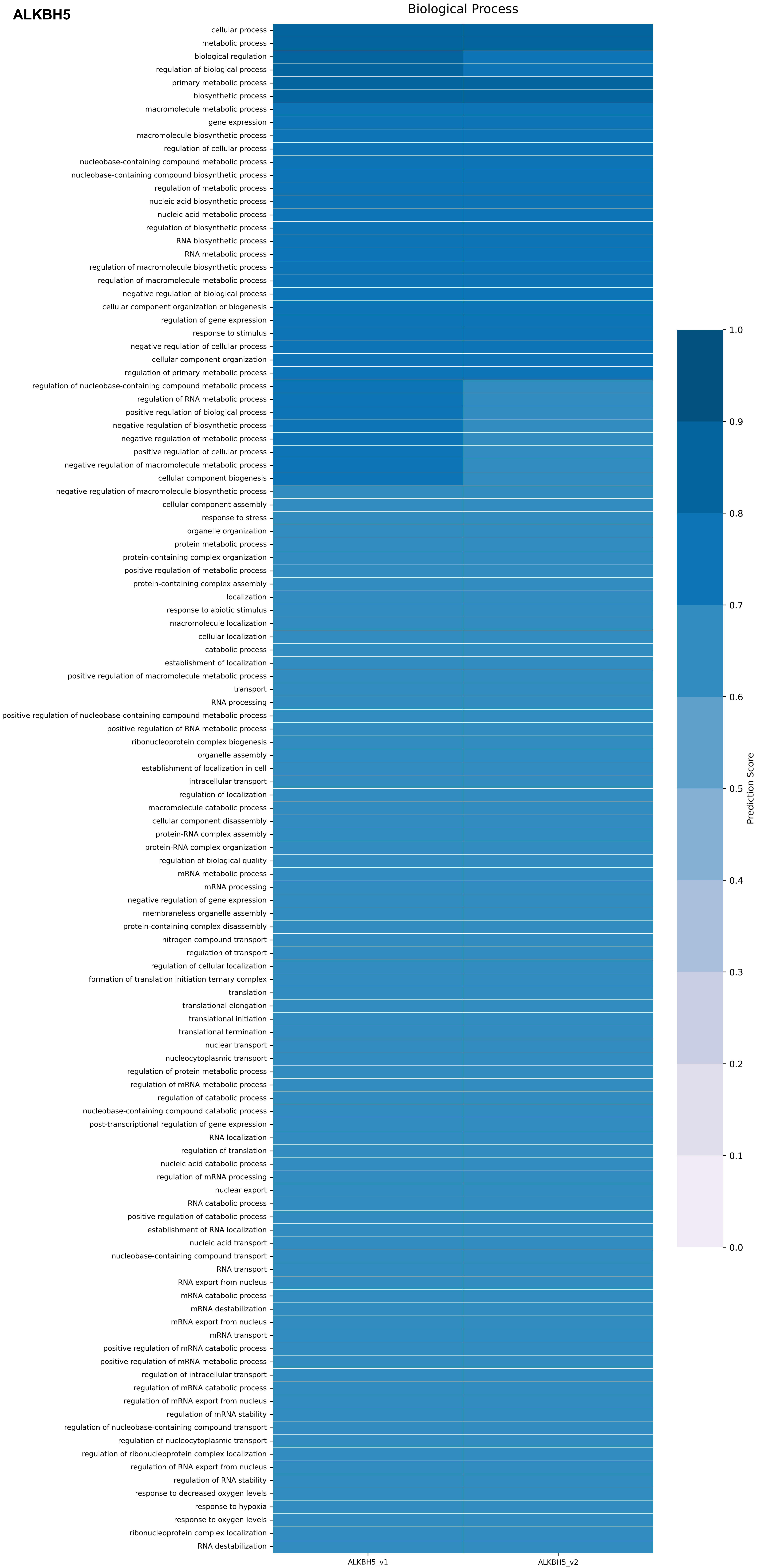
ALKBH5
Biological Process
cellular process
metabolic process
biological regulation
regulation of biological process
primary metabolic process
biosynthetic process
macromolecule metabolic process
gene expression
macromolecule biosynthetic process
regulation of cellular process
nucleobase-containing compound metabolic process
nucleobase-containing compound biosynthetic process
regulation of metabolic process
nucleic acid biosynthetic process
nucleic acid metabolic process
regulation of biosynthetic process
RNA biosynthetic process
RNA metabolic process
regulation of macromolecule biosynthetic process
regulation of macromolecule metabolic process
negative regulation of biological process
cellular component organization or biogenesis
regulation of gene expression
response to stimulus
negative regulation of cellular process
cellular component organization
regulation of primary metabolic process
regulation of nucleobase-containing compound metabolic process
regulation of RNA metabolic process
positive regulation of biological process
negative regulation of biosynthetic process
negative regulation of metabolic process
positive regulation of cellular process
negative regulation of macromolecule metabolic process
cellular component biogenesis
negative regulation of macromolecule biosynthetic process
cellular component assembly
response to stress
organelle organization
protein metabolic process
protein-containing complex organization
positive regulation of metabolic process
protein-containing complex assembly
localization
response to abiotic stimulus
macromolecule localization
cellular localization
catabolic process
establishment of localization
positive regulation of macromolecule metabolic process
transport
RNA processing
positive regulation of nucleobase-containing compound metabolic process
positive regulation of RNA metabolic process
ribonucleoprotein complex biogenesis
organelle assembly
establishment of localization in cell
intracellular transport
regulation of localization
macromolecule catabolic process
cellular component disassembly
protein-RNA complex assembly
protein-RNA complex organization
regulation of biological quality
mRNA metabolic process
mRNA processing
negative regulation of gene expression
membraneless organelle assembly
protein-containing complex disassembly
nitrogen compound transport
regulation of transport
regulation of cellular localization
formation of translation initiation ternary complex
translation
translational elongation
translational initiation
translational termination
nuclear transport
nucleocytoplasmic transport
regulation of protein metabolic process
regulation of mRNA metabolic process
regulation of catabolic process
nucleobase-containing compound catabolic process
post-transcriptional regulation of gene expression
RNA localization
regulation of translation
nucleic acid catabolic process
regulation of mRNA processing
nuclear export
RNA catabolic process
positive regulation of catabolic process
establishment of RNA localization
nucleic acid transport
nucleobase-containing compound transport
RNA transport
RNA export from nucleus
mRNA catabolic process
mRNA destabilization
mRNA export from nucleus
mRNA transport
positive regulation of mRNA catabolic process
positive regulation of mRNA metabolic process
regulation of intracellular transport
regulation of mRNA catabolic process
regulation of mRNA export from nucleus
regulation of mRNA stability
regulation of nucleobase-containing compound transport
regulation of nucleocytoplasmic transport
regulation of ribonucleoprotein complex localization
regulation of RNA export from nucleus
regulation of RNA stability
response to decreased oxygen levels
response to hypoxia
response to oxygen levels
ribonucleoprotein complex localization
RNA destabilization
ALKBH5_v1
ALKBH5_v2
Prediction Score
1.0
0.9
0.8
0.7
0.6
0.5
0.4
0.3
0.2
0.1
0.0

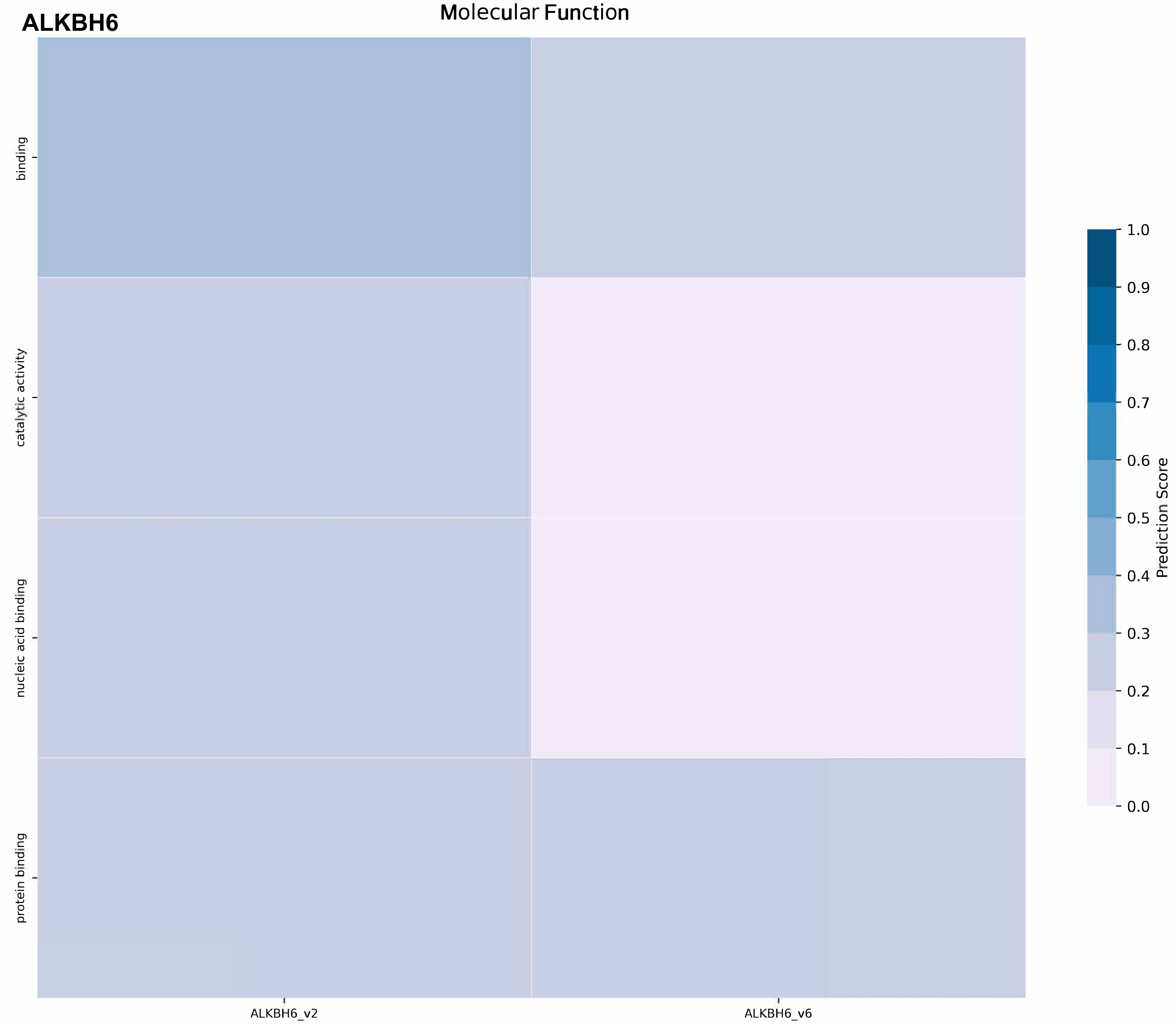

ALKBH6
Molecular Function
binding
catalytic activity
nucleic acid binding
protein binding
ALKBH6_v2
ALKBH6_v6
Prediction Score
1.0
0.9
0.8
0.7
0.6
0.5
0.4
0.3
0.2
0.1
0.0

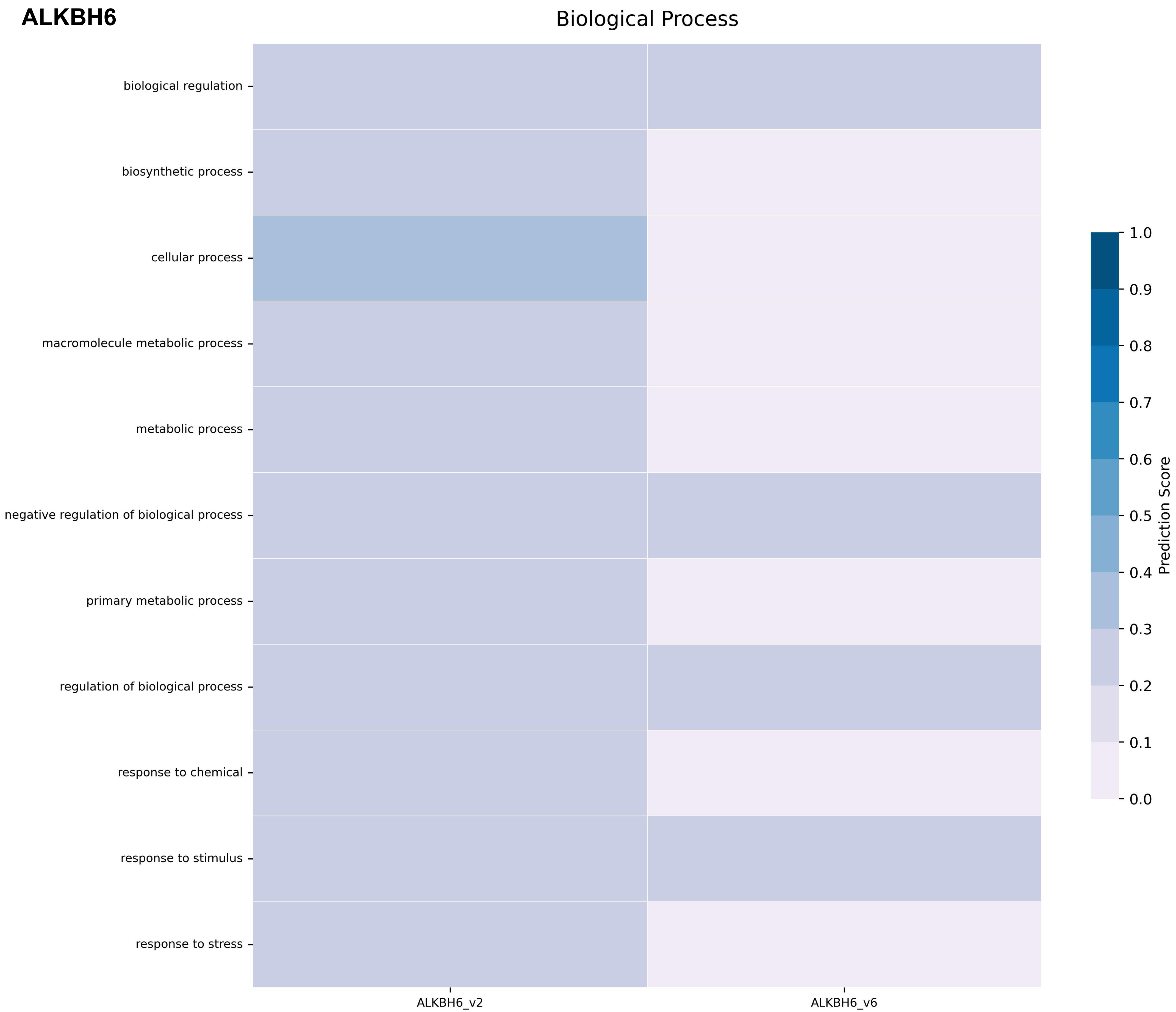
ALKBH6
Biological Process
biological regulation
biosynthetic process
cellular process
macromolecule metabolic process
metabolic process
negative regulation of biological process
primary metabolic process
regulation of biological process
response to chemical
response to stimulus
response to stress
ALKBH6_v2
ALKBH6_v6
Prediction Score
0.0
0.1
0.2
0.3
0.4
0.5
0.6
0.7
0.8
0.9
1.0

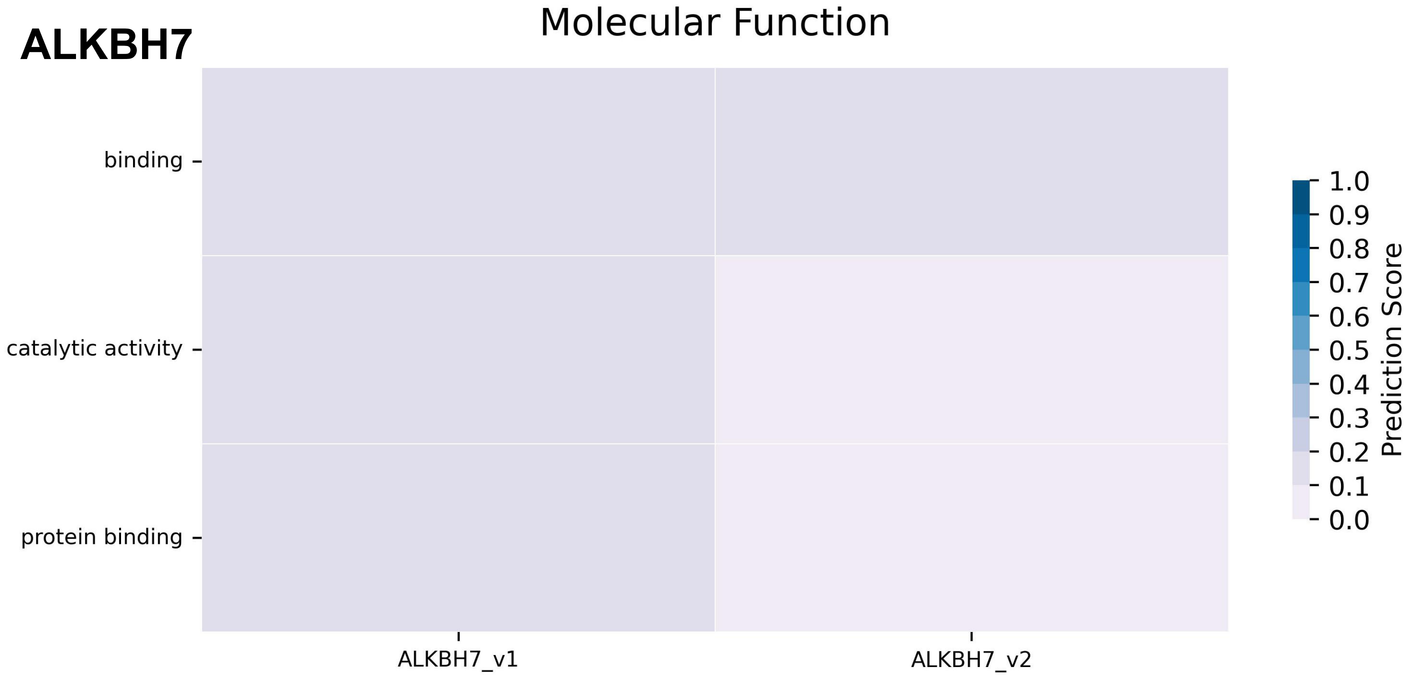
ALKBH7
Molecular Function
binding
catalytic activity
protein binding
ALKBH7_v1
ALKBH7_v2
1.0
0.9
0.8
0.7
0.6
0.5
0.4
0.3
0.2
0.1
0.0
Prediction Score

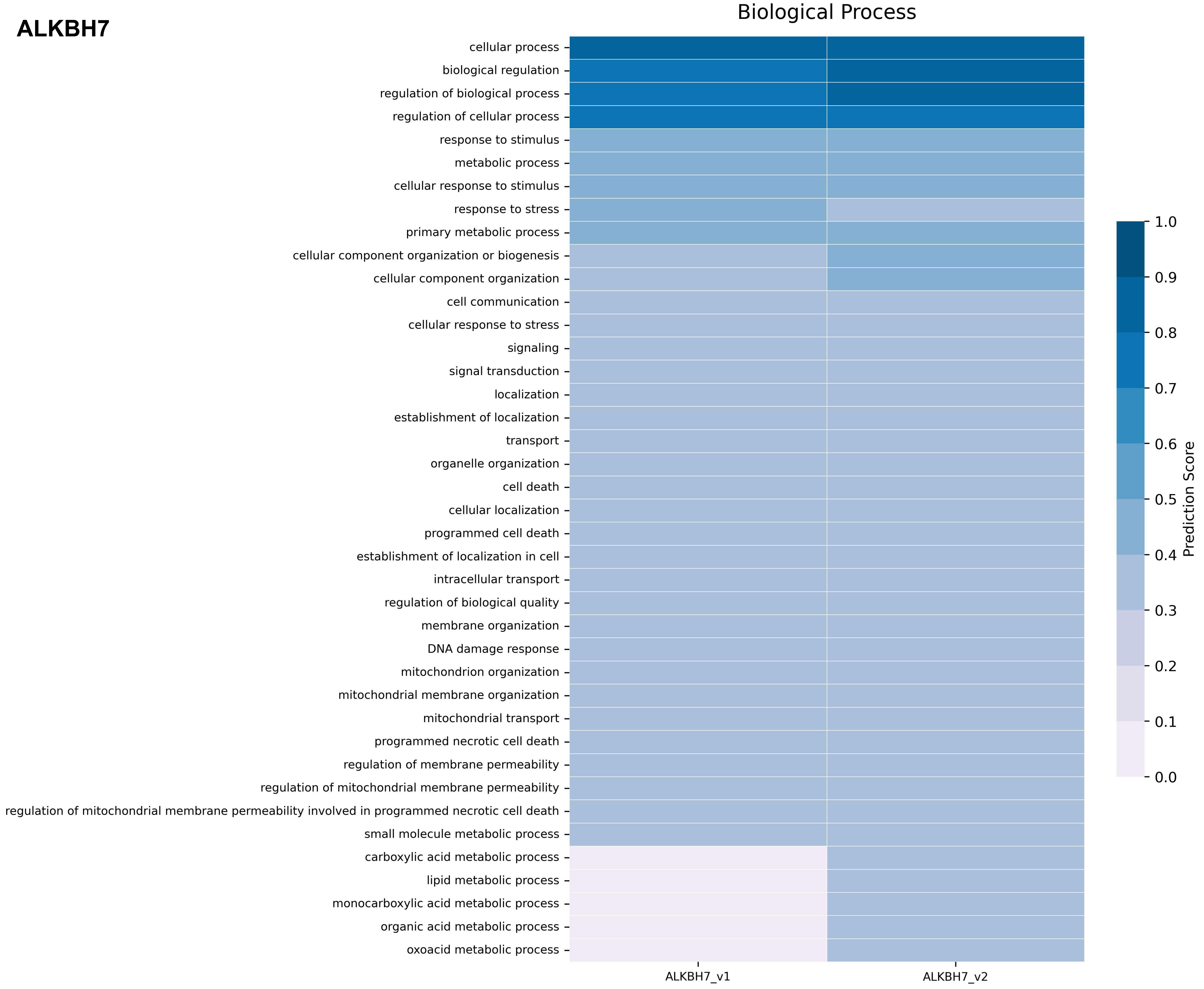
ALKBH7
Biological Process
cellular process
biological regulation
regulation of biological process
regulation of cellular process
response to stimulus
metabolic process
cellular response to stimulus
response to stress
primary metabolic process
cellular component organization or biogenesis
cellular component organization
cell communication
cellular response to stress
signaling
signal transduction
localization
establishment of localization
transport
organelle organization
cell death
cellular localization
programmed cell death
establishment of localization in cell
intracellular transport
regulation of biological quality
membrane organization
DNA damage response
mitochondrion organization
mitochondrial membrane organization
mitochondrial transport
programmed necrotic cell death
regulation of membrane permeability
regulation of mitochondrial membrane permeability
regulation of mitochondrial membrane permeability involved in programmed necrotic cell death
small molecule metabolic process
carboxylic acid metabolic process
lipid metabolic process
monocarboxylic acid metabolic process
organic acid metabolic process
oxoacid metabolic process
ALKBH7_v1
ALKBH7_v2
Prediction Score
1.0
0.9
0.8
0.7
0.6
0.5
0.4
0.3
0.2
0.1
0.0
