# Supplementary material for: Identification of Novel Alternative Transcripts of the Human ALKBH Gene Family and Investigation of Their Unique Expression Signatures in Cancer Cells
Source: Curr Issues Mol Biol. 2026 Feb 26;48(3):251. doi: 10.3390/cimb48030251 (PMC13026028; doi:10.3390/cimb48030251)
Supplement: Supplementary file 1 [file cimb-48-00251-s001.zip › Supplementary Figure Legends.pdf]

Supplementary Figure Legends.

Supplementary Figure S1. Protein models of the described putative ALKBH3 isoforms generated using AlphaFold3. Distinct colors highlight the conserved structural motifs within the ALKBH3 proteins. For each protein isoform, only the 3D structure with the highest confidence score is demonstrated.

Supplementary Figure S2. Structural demonstration of the ALKBH4 proteins obtained by AlphaFold3 software. Different colors are used to indicate the conserved motifs of ALKBH4. Only the highest-confidence model for each isoform is displayed.

Supplementary Figure S3. Predicted 3D structure models of the ALKBH5 proteins. Each domain is shown in different color. For each isoform, only the 3D structure with the highest confidence score is demonstrated.

Supplementary Figure S4. AlphaFold3-generated models illustrate the structural organization of ALKBH6 isoforms. Conserved motifs are highlighted in distinct colors. For each isoform, only the highest-confidence model is presented.

Supplementary Figure S5. Predicted 3D structure models of ALKBH7 isoforms. Each domain is color-coded, and for each isoform, only the model with the highest confidence score is shown.
