# Supplementary material for: Identification of Novel Alternative Transcripts of the Human ALKBH Gene Family and Investigation of Their Unique Expression Signatures in Cancer Cells
Source: Curr Issues Mol Biol. 2026 Feb 26;48(3):251. doi: 10.3390/cimb48030251 (PMC13026028; doi:10.3390/cimb48030251)
Supplement: Supplementary file 1 [file cimb-48-00251-s001.zip › Supplementary Table S1.pdf]

Supplementary Table S1. Panel of cell lines used in this study and their corresponding tissue of origin.

Cell type	Cell line
Breast/ductal adenocarcinoma	BT-20
Ovarian cancer	OVCAR-3
Prostate cancer	PC-3
Colorectal cancer	HCT 116
Cervical adenocarcinoma	HeLa
Hepatocellular carcinoma	HepG2
Acute monocytic leukemia	THP-1
Osteosarcoma	U-2 OS
Urinary bladder carcinoma	RT-112
Non-cancerous cell lines	HEK-293, MCF 10A
