# Supplementary material for: Identification of Novel Alternative Transcripts of the Human ALKBH Gene Family and Investigation of Their Unique Expression Signatures in Cancer Cells
Source: Curr Issues Mol Biol. 2026 Feb 26;48(3):251. doi: 10.3390/cimb48030251 (PMC13026028; doi:10.3390/cimb48030251)
Supplement: Supplementary file 1 [file cimb-48-00251-s001.zip › Supplementary Table S2.pdf]

Supplementary Table S2. List of primers that were used for the specific qPCR amplification of mRNAs of the *ALKBH* gene family. The melting temperature (T_m) for each primer was calculated by Primer-BLAST designing tool.

Genes	Transcripts	Name	Sequence (5′→3′)	Length (nt)	T_m (°C)
ALKBH1	v.1	Ex3/4F	GTTCCTGAGGTATAAAGAAGCGA	24	59.2
		Ex5/6R	CTGTCCAAAGCTGAATGACAGC	22	60.1
	v.2	Ex2/4F	GGCTATCCTGGTATAAAGAAGCG	23	58.75
		Ex4/alt5R	CGGCTACTTGCTCTTACTGTCC	22	60.74
ALKBH2	v.1	1/2F	GGCAGCGAGGACATGGTTTT	20	61.24
		2/3R	GGCCAGTGCTCCTGTAAAATATTC	24	59.96
	v.6	1/inF	GGCAGCGAGGTCAGTTATGG	20	60.81
		in/2R	CCATGTCCTAGAAAGGAAACAG	22	56.43
ALKBH3	v.1	Ex5/6F	CCACAGGTGTATCTAGGGTCTGT	23	60.88
		Ex7/8R	CAGGGTGCCAGTGAGGATTTG	21	61.22
	v.2	Ex5/6F	CCACAGGTGTATCTAGGGTCTGT	23	60.88
		Ex6/8R	CAGGGTGCCACCTCTCTGAT	20	61.27
	v.3	Ex5/6F	CCACAGGTGTATCTAGGGTCTGT	23	60.88
		Ex6/9R	CTCCATTCTCTTCCCTCTCTGATG	24	59.96
	v.4	Ex5/6F	CCACAGGTGTATCTAGGGTCTGT	23	60.88
		Ex7/9R	AGTCTCCATTCTCTTCGTGAGGA	23	60.56
ALKBH4	v.1	Ex1/2F	CTGCCCCCAGCGAAAACATA	20	60.68
		Ex2/3R	GCCATAGTCCTGCTTCCTCC	20	59.89
	v.2	Ex1/3F	TGCCCCCAGCGGACTATG	18	61.48
		Ex3R	CAGGTTGCACTGCTCGACG	19	61.66
ALKBH5	v.1	Ex1/2F	GACTGTGCTCAGTGGATATGCT	22	60.16
		Ex3/4R	CAGGATCCGTGGCCTGTG	18	60.12
	v.2	Ex1/2F	GACTGTGCTCAGTGGATATGCT	22	60.16
		Ex2/4R	AGGATCCGTGGCTTCCTGAG	20	61.33
ALKBH6	v.2	Ex4/5F	TTACAGAACTGGGGTGGGCT	20	60.77
		Ex5R	CAGGCAGATACTGGTTCACGAG	22	60.74
	v.6	Ex4/6F	TTACAGAACTGGGCCCCACGA	21	63.47
		Ex6/7R	AGGCCGAGGCTGTTCTGTAG	20	61.61
ALKBH7	v.1	Ex2/3F	GTGGACAGCATCAAGTTCTGC	21	59.8
		Ex3/4R	GGCTGAGCCCCTAAGGATGT	20	61.35
	v.2	Ex3/inF	CCCTCTACATCCTTAGGTACCTCC	24	60.75
		in/4R	CTGAGCCCCTGCAGGAAGAA	20	61.85
