# Supplementary figures and images for: Identification of Novel Alternative Transcripts of the Human ALKBH Gene Family and Investigation of Their Unique Expression Signatures in Cancer Cells
Source: Curr Issues Mol Biol. 2026 Feb 26;48(3):251. doi: 10.3390/cimb48030251 (PMC13026028; doi:10.3390/cimb48030251)

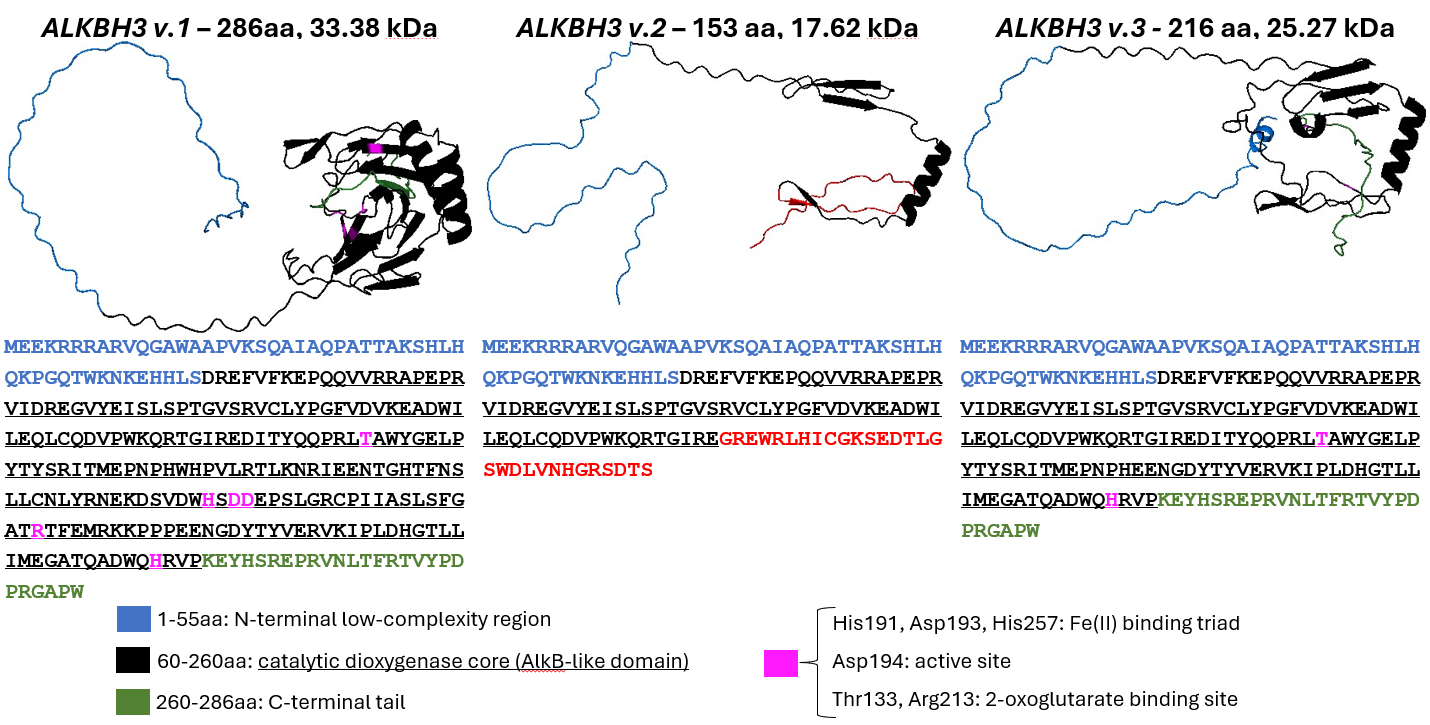

Supplement: Supplementary file 1 [file cimb-48-00251-s001.zip › Supplementary Figure S1.tif]

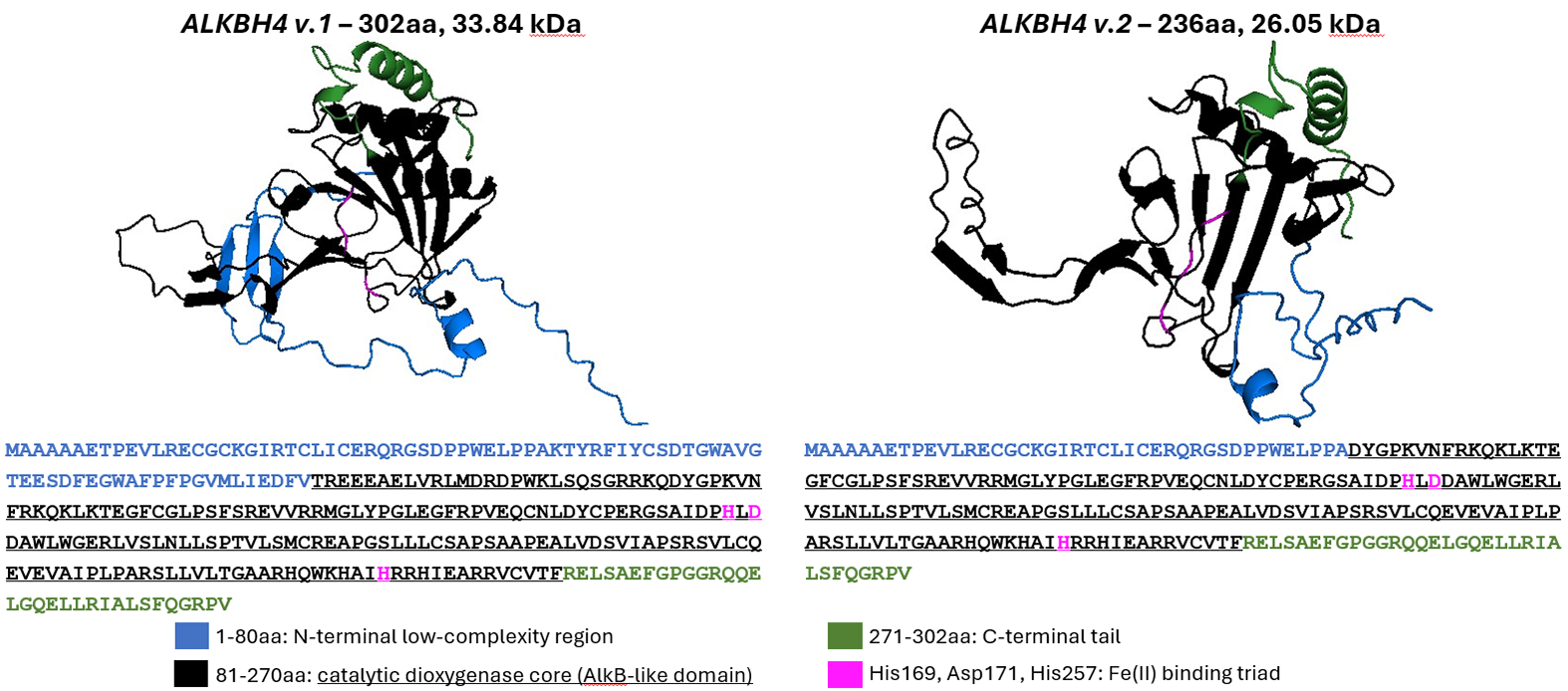

Supplement: Supplementary file 1 [file cimb-48-00251-s001.zip › Supplementary Figure S2.tif]

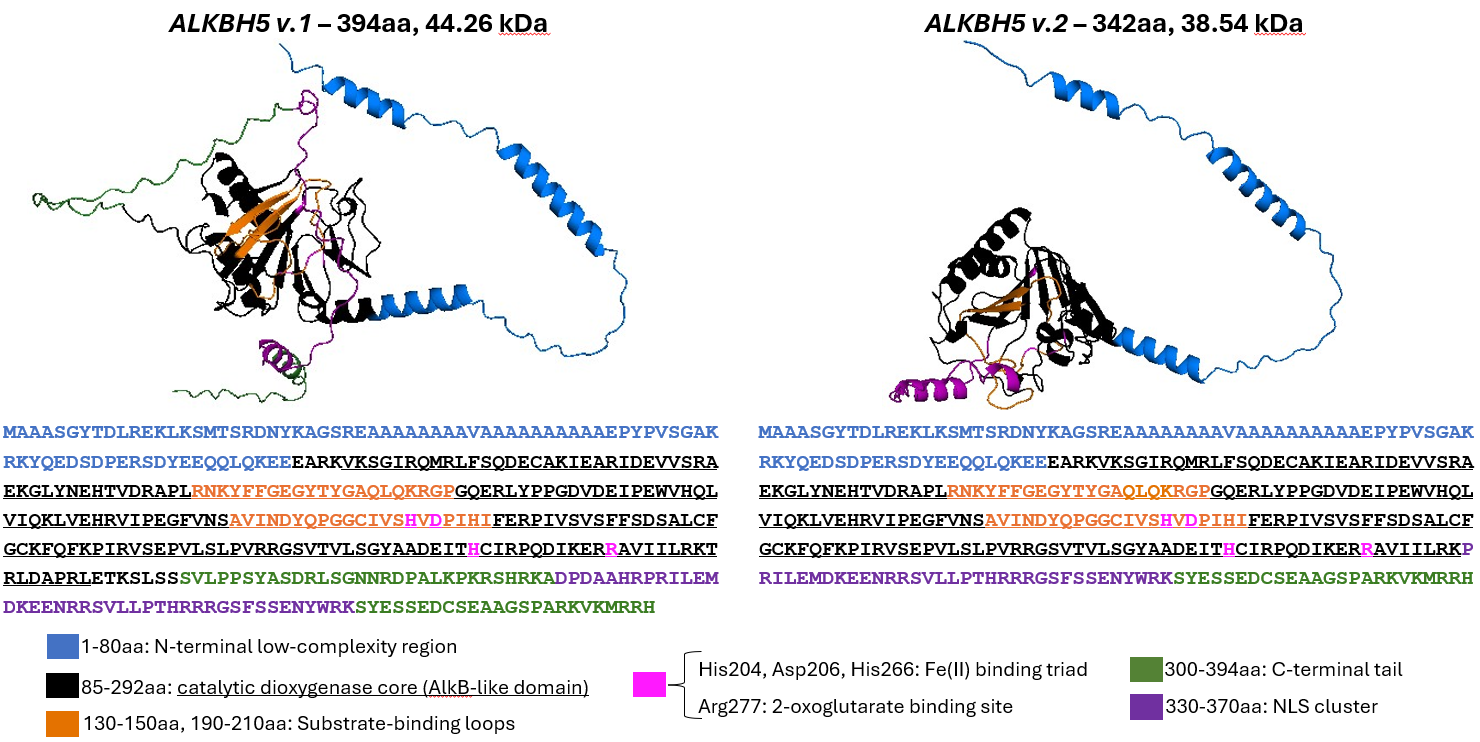

Supplement: Supplementary file 1 [file cimb-48-00251-s001.zip › Supplementary Figure S3.tif]

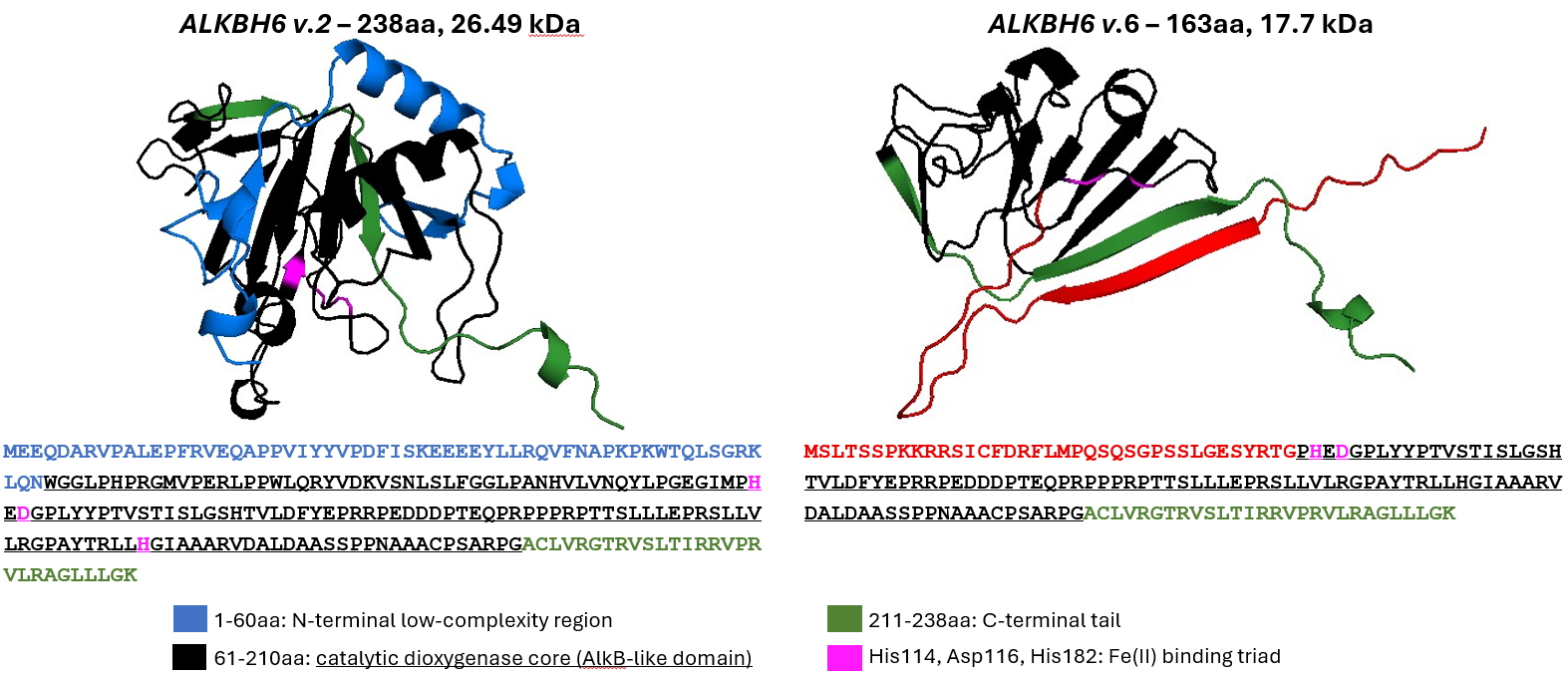

Supplement: Supplementary file 1 [file cimb-48-00251-s001.zip › Supplementary Figure S4.tif]

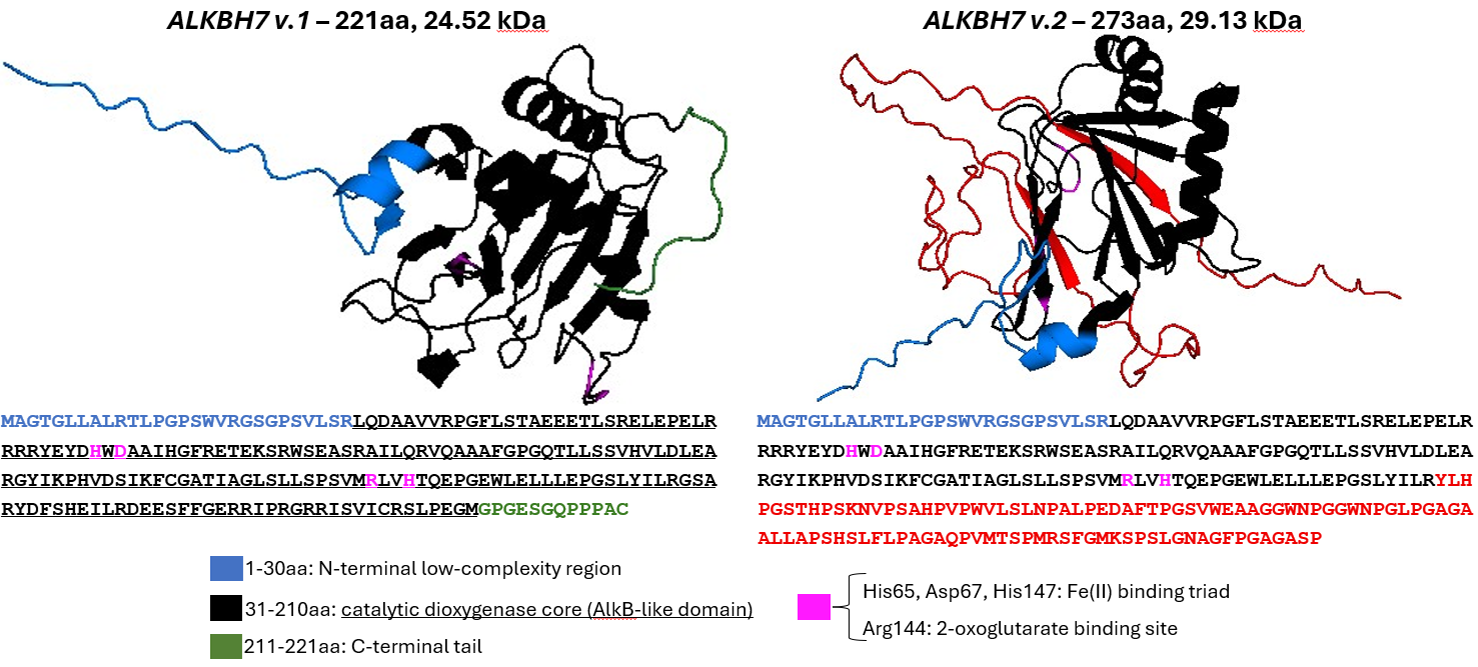

Supplement: Supplementary file 1 [file cimb-48-00251-s001.zip › Supplementary Figure S5.tif]
